# Identification of catalytically important residues of the carotenoid 1,2-hydratases from *Rubrivivax gelatinosus* and *Thiocapsa roseopersicina*

**DOI:** 10.1007/s00253-015-6998-y

**Published:** 2015-10-19

**Authors:** Aida Hiseni, Linda G. Otten, Isabel W. C. E. Arends

**Affiliations:** Biocatalysis Group, Department of Biotechnology, Delft University of Technology, Julianalaan 67, 2628 BC Delft, The Netherlands

**Keywords:** Carotenoid 1,2-hydratase, CrtC, Hydro-lyase, Enzyme mechanism, Biocatalysis

## Abstract

Carotenoid 1,2-hydratases (CrtC) catalyze the selective addition of water to an isolated carbon–carbon double bond. Although their involvement in the carotenoid biosynthetic pathway is well understood, little is known about the mechanism by which these hydratases transform carotenoids such as lycopene into the corresponding hydroxyl compounds. Key residues were identified at positions His239, Trp241, Tyr266, and Asp268 in CrtC from *Rubrivivax gelatinosus* (and corresponding positions in *Thiocapsa roseopersicina*). Alanine mutants at these positions were found to be completely inactive, suggesting their direct involvement in the catalytic reaction. Our resulting mechanistic hypothesis is in analogy with the recently studied class of terpenoid cyclase enzymes containing a highly acidic aspartic residue in their active site. We propose that a similar aspartic acid residue, which is conserved through all putative CrtCs, is involved in initial protonation of the double bond in lycopene.

**Electronic supplementary material:**

The online version of this article (doi:10.1007/s00253-015-6998-y) contains supplementary material, which is available to authorized users.

## Introduction

Carotenoids, which represent one of the most abundant natural pigments with structural and protective properties (Armstrong and Hearst 1996), play an essential role in the photosynthetic machinery of phototrophic organisms such as purple bacteria (Jensen et al. 1961) and higher plants (Cazzonelli 2011). In addition, they have been identified in fungi and some non-photosynthetic bacteria (Armstrong 1997). Carotenoid 1,2-hydratase (also known as CrtC) is a member of hydro-lyase group EC 4.2.1.131 (Hiseni et al. 2015). The enzyme takes part in the biosynthetic pathway of carotenoids (Umeno et al. 2005). CrtC introduces a tertiary hydroxyl group into an acyclic carotenoid molecule by addition of water to the carbon–carbon double bond at the C-1 position. The enzyme belongs to Pfam family PF07143 that encompasses members from several purple photosynthetic bacteria. On the other hand, CrtCs have been identified, which are able to hydrate monocyclic carotenoid gamma-carotene. These are evolutionary very distinct from the PF07143 members and have been given the name CruF (Sun et al. 2009).

**Fig. 1:**
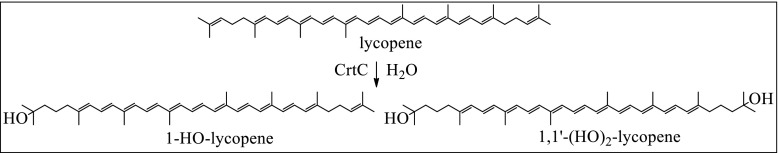
Reaction catalyzed by *Rubrivivax gelatinosus* and *Thiocapsa roseopersicina* carotenoid 1,2-hydratase; the conversion of lycopene into 1-HO-lycopene and 1,1′-(HO)_2_-lycopene

To date, two representatives of the PF07143 family, the CrtCs from purple non-sulfur Betaproteobacteria *Rubrivivax gelatinosus* and purple sulfur Gammaproteobacteria *Thiocapsa roseopersicina*, were recombinantly expressed and characterized (Hiseni et al. 2011). Biochemical studies have revealed that these enzymes are able to convert cofactor-independently lycopene into 1-HO-lycopene and 1,1′-(HO)_2_-lycopene (Fig. 1). In addition, they showed some activity towards the unnatural substrate geranylgeraniol, a C20 molecule that resembles the natural substrate lycopene.

CrtCs are appealing enzymes in the biotechnology field because they are able to generate a tertiary alcohol, a highly valuable building block for the synthesis of several bioactive natural products and pharmaceuticals (Hiseni et al. 2015; Kourist et al. 2008). Furthermore, they possess an intrinsically high stability at a wide pH and temperature range, which constitute useful properties for an industrial application (Hiseni et al. 2011). The subcellular location of this enzyme in the cell membrane fraction (membrane associated) allows for a straightforward isolation and simplified large-scale purification.

From a chemical point of view, CrtCs are able to perform a challenging chemical reaction, namely the selective addition of water to an isolated carbon–carbon double bond (Jin and Hanefeld 2011). Using the enzyme, the reaction proceeds without assistance of electron-withdrawing groups or transition metal cations, while the chemical hydration requires harsh acidic conditions (Evans and Kirby 1984). Furthermore, the CrtCs from photosynthetic bacteria act on acyclic carotenoids, whereas the CruFs from non-photosynthetic bacteria catalyze the hydration of monocyclic carotenoids. To our knowledge, no published data exist on the catalytic and structural features that determine hydratase activity and specificity of these two distinct families, nor has the 3D structure been elucidated yet. The mechanism of lycopene hydration, which involves proton attack at C-2 and C-2′ and the introduction of the hydroxyl group at C-1 and C-1′, was established from ^2^H_2_O and H_2_^18^O labeling studies with intact cells (Patel et al. 1983; Yeliseev and Kaplan 1997). For a hydration reaction, it is likely to assume that the first step in the reaction is protonation of the alkene, leading to an intermediate carbocation. Quenching of the carbocation by water will lead to the alcohol as product. The protonation of hydrophobic long-chain alkenes has also been described for the enzyme class of cyclases, of which the full mechanism is known (Hammer et al. 2013; Wendt et al. 2000).

The objective of this study was to provide insight into the hydration mechanism of CrtCs. This knowledge is pivotal in order to engineer this promising enzyme class towards, i.e., higher activities, better stability, or widening of its substrate scope. Through multi-sequence alignment of several CrtC homologues, highly conserved amino acids were identified, which could be functionally or structurally important. The corresponding alanine mutants of these amino acids were produced to evaluate their involvement in the hydratase activity. Following the identification of probable catalytically active amino acid residues, the aim was to propose a catalytic mechanism for addition of water catalyzed by CrtC.

## Materials and methods

### In silico analysis

Basic Local Alignment Search Tool (BLAST) (Altschul et al. 1990) was used to find and select carotenoid 1,2-hydratase homologues of CrtC from *R. gelatinosus* (*Rg*CrtC) using default settings; e.g., for nucleotide BLAST, the tblastn option was used with the nucleotide database (nt/nr), and for protein BLAST, the blastp option was used with the non-redundant protein database (nr). In order to look for identities/similarities between the CrtC homologues, nucleotide and amino acid sequences were aligned with the BioEdit Sequence Alignment Editor v.7.1.3.0 (www.mbio.ncsu.edu/bioedit/bioedit.html) or ClustalW (Larkin et al. 2007). In addition, protein sequences were subjected to protein functional analysis using a search in the Conserved Domain Database (CDD) (Marchler-Bauer et al. 2011) and Pfam search (Finn et al. 2010), using the standard parameters on the respective websites of these tools. A protein phylogenetic tree was constructed with Phylogeny.fr using the “One Click” program settings (Dereeper et al. 2010; Dereeper et al. 2008). These settings represent a *default* mode which proposes a pipeline already set up to run and connect programs recognized for their accuracy and speed to reconstruct a robust phylogenetic tree from a set of sequences (MUSCLE for multiple alignment, optionally Gblocks for alignment curation, PhyML for phylogeny, and finally, TreeDyn for tree drawing).

### Cloning of carotenoid 1,2-hydratase genes

Plasmids pET15b_CrtC_Rg_ and pET15b_CrtC_Tr_ containing CrtC from *R. gelatinosus* (*Rg*) and *T. roseopersicina* (*Tr*), respectively, were constructed in a previous study (Hiseni et al. 2011). Two fosmids with *crtC* genes from metagenomic samples DelRiverFos06H03 (Fos06) and DelRiverFos13D03 (Fos13), respectively, were kindly provided by Dr. Kirchman (Waidner and Kirchman 2005). The cosmid encoding CrtC from *Bradyrhizobium* (*Br*) was received from Dr. Dreyfus (Giraud et al. 2004). In order to get sufficient DNA material for further studies, the fosmid DNA and cosmid DNA were amplified in *Escherichia coli* TOP10 cells. After DNA isolation with the QIAprep Spin Miniprep Kit (Qiagen) from the cells, sufficient DNA was obtained for further research. The *crtC*s from *Rhodospirillum rubrum* (*Rr*) and *Rhodopseudomonas palustris* (*Rp*) were amplified from genomic DNA. For that, genomic DNA of *R. rubrum* (*Rr*) was kindly provided by Prof. Roberts (NCBI Reference Sequence: NC_007641.1). *R. palustris* cells (DSM No. 123) were obtained from DSMZ (Deutsche Sammlung von Mikroorganismen und Zellkulturen), enriched in appropriate medium according to DSMZ instructions and gDNA isolated using the UltraClean Soil DNA Isolation Kit (Mo Bio). Subsequently, primers were designed for the isolation of all *crtC* genes (Table 1), which carry two restriction sites for subsequent cloning: *Nde*I (forward) and *Xho*I (reverse). For *BrcrtC***,** the *Xho*I site was replaced with *Bam*HI, because the *Xho*I site was present in the gene itself. Amplification reactions were done using standard PCR reactions. Using the appropriate enzymes, the fragment was digested, purified**,** and ligated into the same sites of the pET15b vector and transformed into *E. coli* TOP10 competent cells. The insertion of the *crtC* gene was verified by restriction analysis with the corresponding restriction enzymes (New England Biolabs) and DNA sequencing (BaseClear, Leiden, The Netherlands).

**Table 1:** Primers used for PCR and subsequent cloning of the genes into the expression vector pET15b

Name	Sequence (5′→3′)	Restriction site
DRF06 FW	GGGAGTACCATATGAGTGATGATGGCCAAC	*Nde*I
DRF06 RV	ATCCGCTCGAGATAATCTCAAGCCCGCCTCG	*Xho*I
DRF13 FW	GGGAGTACATATGGATGGCGTGTCAGAC	*Nde*I
DRF13 RV	CCGCTCGAGTAATGCTTAGGGCCACTTGGC	*Xho*I
Br FW	CGGACATCATATGTGCCCGCCAG	*Nde*I
Br RV	ATCCAGGATCCATCGCGTGAACTTCACCACC	*Bam*HI
Rp FW	CGGGACTTCCATATGTCAGGAGCTGAGTTG	*Nde*I
Rp RV	ACCGCTCGAGTAACGTTCAGCGGAACGC	*Xho*I
Rr FW	GGGAAATTCCATATGCACCGCCCGGAC	*Nde*I
Rr RV	GCTCGAGTTCAATTAGCCCTTAACCGCCGC	*Xho*I

The respective restriction sites are underlined

### Single point mutations

Single amino acid exchange within the *crtC* genes of *Rg* and *Tr* was done using the megaprimer PCR method introduced by Kammann et al. (1989) and later modified by Sarkar and Sommer (1990) and Landt et al. (1990). The mismatch primers are listed in Table 2. In the first PCR reaction, performed under standard reaction conditions, the megaprimer was produced using the corresponding forward primer containing the desired base substitution (Table 2) in combination with the reverse primer Rg_rv or Tr_rv (Hiseni et al. 2011). Plasmids pET15b_CrtC_Rg_ and pET15b_CrtC_Tr_ (Hiseni et al. 2011) were used as the template. The size and purity of the megaprimer was verified by agarose gel electrophoresis. In order to produce the full-length gene, a second PCR reaction was performed with the corresponding megaprimer and *Rg*_fw or *Tr*_fw (Hiseni et al. 2011). Subsequent steps were performed as described in the previous section. The presence of the desired mutation was verified by DNA sequencing (BaseClear, Leiden, The Netherlands).

**Table 2:** Primers for site-directed mutagenesis

	Amino acid exchange	Sequence (5′→3′)
*R. gelatinosus*	H239A	AGCGGCGGACGCGCTCGCTG
	W241A	CATCGCGCGGGGCCGATCG
	H264A	CTGGAGCGGCGCCGCCTACC
	Y266A	GCCACGCCGCCCTCGACT
	D268A	CGCCTACCTCGCCTCGAACGAAG
*T. roseopersicina*	H237A	GATCCGGCGGAACGCGCAGTCTGGTGG
	W239A	CGCCATGTCGCGTGGCCGATC
	H262A	GCTGGAGCGGCGCTGGCTAT
	D266A	CATGGCTATCTCGCCTCAAA
	S58V	GCGTCCGTCGTCGCGCAGCA
	S58Q	GCGTCCCAGGTCGCGCAGCA

Mismatch points are underlined

### N-terminally truncated *Rg*CrtC and *Tr*CrtC

*Rg*CrtC and *Tr*CrtC lacking the first 45 and 57 amino acids, respectively, were constructed using primers Rg_45aa/Rg_rv and Tr_57aa/Tr_rv (Table 3; Hiseni et al. 2011) under standard PCR conditions. Plasmids pET15b_CrtC_Rg_ and pET15b_CrtC_Tr_ (Hiseni et al. 2011), respectively, were used as the template. Subsequent steps including verification were performed as described in the “Cloning of carotenoid 1,2-hydratase genes” section.

**Table 3:** Primers used for construction of truncated CrtCs

Name	Sequence (5′→3′)
Rg_45aa	AGTACCATATGGGCGACGCACGGCTGG
Tr_57aa	AGTACCATATGTCCGTCGCGCAGCAAGG

The *Nde*I restriction sites are underlined

### Recombinant expression of CrtCs

*E. coli* BL21 (DE3) was the host for the pET15_CrtC plasmids. Cultures were grown at 37 **°**C in Luria–Bertani broth with 100 μg ml^−1^ ampicillin until an OD600 value of 0.6–0.8 was reached. Unless otherwise stated, protein expression was induced by addition of isopropyl-β-d-thiogalactopyranoside (IPTG) to a final concentration of 0.1 mM, followed by cultivation at 25 **°**C overnight. The cells were harvested by centrifugation at 10,000 rpm for 10 min at 4 **°**C (Sorvall), washed once with 50 mM Na_2_HPO_4_ buffer (pH 8.0), and suspended in the same buffer. In case of subsequent purification, 10 mM imidazole was added to the buffer. Crude extract (CE) from cultures >100 ml was prepared by adding 1 mg ml^−1^ lysozyme and incubating the cells for 1 h at 4 **°**C, followed by cell disruption at the pressure of 1.5 kBar (Constant Systems; IUL Instruments). For cultures <100 ml, the cells were disrupted by sonication for 2 min while immersed in an ice-water bath using the microtip probe of a sonicator (Branson Sonicator Cell Disruptor) set at 50 % maximal energy. In an effort to reduce the liquid viscosity caused by DNA molecules, 0.1 mg ml^−1^ of DNAse was added. With the subsequent centrifugation at 10,000 rpm for 20 min at 4 **°**C, cell-free extract (CFE) and pellet were separated. Protein content of the crude extract was determined by BCA assay (Pierce) with bovine serum albumin as the reference protein.

### CrtC purification

*Rg*CrtC and *Tr*CrtC “active site” point mutants were purified from the membrane fraction, while the *Tr* “processing” mutants (S58V, S58Q) were purified from the CFE. The membrane fraction was obtained after the centrifugation of the CFE for 4 h at 13,200 rpm and 8 **°**C. The membranes were homogenized by ca. 20 passages through a 25-G needle. Ni-NTA Sepharose HP (GE Healthcare) (previously equilibrated in 50 mM Na_2_HPO_4_ buffer, pH 8.0, 300 mM NaCl, 10 mM imidazole) was added to the CFE or membrane sample. The mixtures were incubated for 1 h at RT, loaded into a polypropylene tube with porous disc (GE Healthcare), and washed three times with washing buffer (50 mM Na_2_HPO_4_ buffer, pH 8.0, 300 mM NaCl, 75 mM imidazole). The CrtC protein was eluted from the column with elution buffer (50 mM Na_2_HPO_4_ buffer, pH 8.0, 300 mM NaCl, 1 M imidazole). Enzyme fractions were separated by sodium dodecyl sulfate–polyacrylamide gel electrophoresis (SDS-PAGE) (10 % Bis-Tris; Bio-Rad) and visualized by staining with SimplyBlue SafeStain (Invitrogen).

### Determination of enzyme activity

Enzymatic activities were determined with CE using lycopene as the substrate according to the method described earlier (Hiseni et al. 2011), with few modifications. The assay was carried out with 50 μl CE and 20 μM substrate, and 10 mg ml^−1^l-α-phosphatidylcholine, in a reaction volume of 200 μl. Prior to the analysis, acetonitrile was added to the reaction mixture in a ratio of 60:40 (ACN/H_2_O), the mixtures were shaken vigorously for 1 min, and solids were removed by centrifugation for 1 min at 13,200 rpm. Separation of the reaction products was performed with a Merck 4.6 × 50 mm Chromolith® SpeedROD RP-18e column with ACN/H_2_O (60:40, *v*/*v*) as the eluent.

## Results

### Comparative in silico analysis of *crtC* genes

**Fig. 2:**
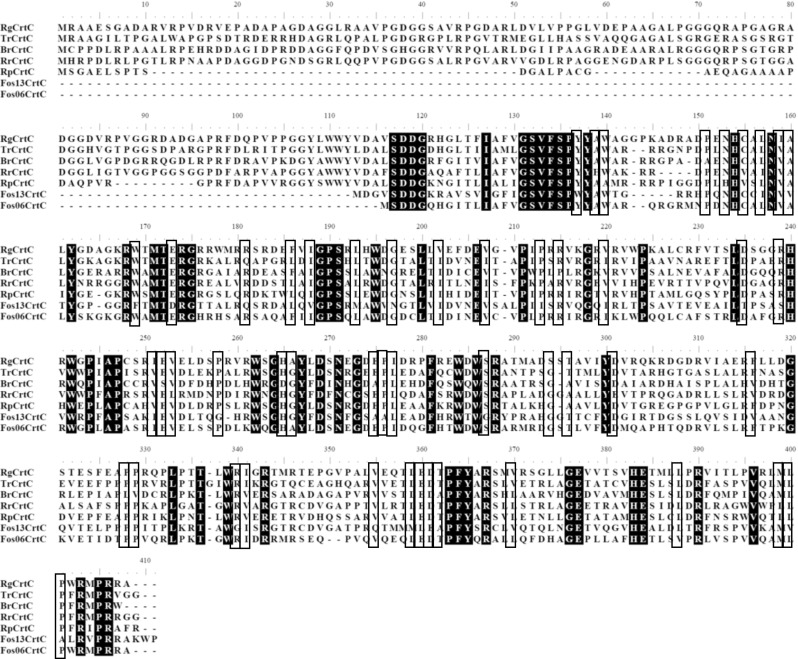
Multiple sequence alignment of CrtC protein sequences probed in this study showing conserved amino acids. Identical amino acids are highlighted in *black*. Positions with only two different amino acids are surrounded by *boxes*

The *Rg-crtC* nucleotide sequence was subjected to a BLAST search in order to identify sequence similarity in different databases. One hundred forty-five hits were identified, of which 119 were representatives of Proteobacteria. BLASTing the protein sequence of *Rg*CrtC resulted in 100 CrtC(-like) sequences, which were aligned in order to investigate if there are any conserved group clusters present (see Electronic Supplementary Material Fig. S1). Indeed, they showed highly conserved regions (Fig. 2, for simplification, the seven sequences used in this study were selected for visualization) distributed along the sequence ranging from amino acid residues ~170 to ~405 (*Rg* numbering). Interestingly, the N-terminal part of the sequence does not contain any conserved amino acids and shows a lot of variation in length, indicating that this region is probably not necessary for CrtC activity. Additionally, we found some blocks of highly conserved residues throughout the C-terminal part of the genes and a cluster of totally conserved residues in the middle of the genes (amino acids 240–280 in *Rg* numbering).

Residues involved in the catalysis tend to be highly conserved in a set of homologous proteins that exhibit the same reaction. On the other hand, sequence insertion and sections of low sequence similarity tend to occur in the less important loop regions (Zvelebil et al. 1987). The conserved blocks in CrtC homologues could indicate that these regions contain the amino acid residues most important for the hydratase activity, specifically those involved in catalysis and substrate binding. From a catalytic point of view, specific amino acids are commonly involved as active residues in acid–base-type catalyzed reactions in the active sites of enzymes (Bartlett et al. 2002). The amino acids aspartic acid (D) or glutamic acid (E) are usually the catalytic acid or base, while tyrosine (Y), tryptophan (W), and histidine (H) typically function as the other part of the charge relay pair (Puthan Veetil et al. 2012). These amino acids are, therefore, the most probable candidates for the catalytic hydration. Four Trp residues, three Tyr residues, and one of each His and Asp residues are fully conserved over the 100 CrtCs (Supplemental Fig. S1). The totally conserved Asp268 (*Rg* numbering) seems to be the most probable candidate for the acid catalysis since it is situated in the middle of the highly conserved region. Furthermore, a fully conserved Tyr residue and a His residue are within close distance, which is important for the contact with the substrate. Fully conserved His and Trp residues are approximately 30 amino acids away but could be in close proximity upon folding of the protein.

### Production of recombinant wild type and mutant CrtCs and enzymatic activity

**Fig. 3:**
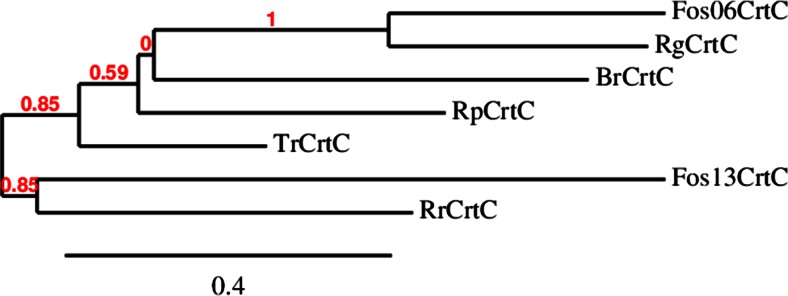
Rooted phylogenetic tree showing the evolutionary relationship between the selected carotenoid 1,2-hydratases. The *scale bar* represents 0.4 substitutions per amino acid site. *Tr*CrtC *Thiocapsa roseopersicina* (GI 31621263), *Br*CrtC *Bradyrhizobium* sp. BTAi1 (GI 146403799), *Fos06*CrtC uncultured Proteobacterium DelRiverFos06H03 (GI 61653228), *Fos13*CrtC uncultured Proteobacterium DelRiverFos13D03 (GI 61653190), *Rp*CrtC *Rhodopseudomonas palustris* (GI 115515977), *Rr*CrtC *Rhodospirillum rubrum* (GI 83574254), *Rg*CrtC *Rubrivivax gelatinosus* (GI 29893477)

**Fig. 4:**
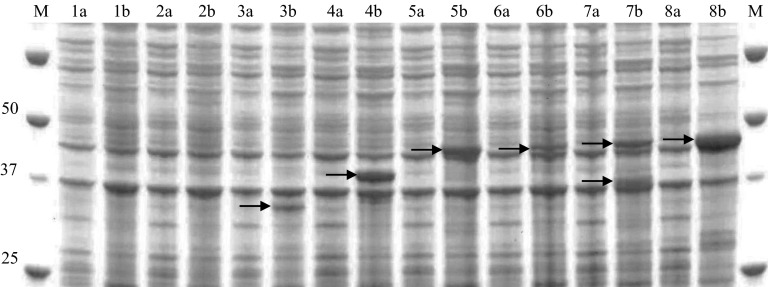
SDS-PAGE (10 %) analysis of CrtC expression in *E. coli* BL21. *M* precision plus protein standard. The *first lane* (*a*) of each sample shows cells before induction with 0.6 mM IPTG, and the *second lane* (*b*) shows cells after 4 h of expression at 37 °C. *1* pET15b control, *2* Fos06CrtC (32 kDa), *3* Fos13CrtC (32 kDa), *4 Rp*CrtC (38 kDa), *5 Rr*CrtC (44 kDa), *6 Br*CrtC (44 kDa), *7 Tr*CrtC (44 kDa), *8 Rg*CrtC (44 kDa). The indicated molecular weights are deduced from amino acid sequences. CrtC expression bands are indicated by *arrows*

Six potential CrtCs were selected for expression and activity studies based on sequence identity with *Rg*CrtC and availability of the corresponding gene constructs. They originate from all three Proteobacteria subclasses (Alphaproteobacteria, Betaproteobacteria, and Gammaproteobacteria) with two additional constructs originating from metagenomic samples from the Delaware River (USA). Figure 3 displays the phylogenetic analysis constructed with protein sequences of the selected CrtC homologues. *Tr*CrtC shows the closest relationship to *Rg*CrtC, followed by *Br*CrtC (55 and 47 % sequence identity, respectively). The combined results of Pfam search and Conserved Domain Database search showed that all seven CrtCs belong to the PF07143 family consisting of several purple photosynthetic bacterial hydroxyneurosporene synthase (CrtC) proteins. Six out of the seven selected CrtCs could be overexpressed from pET15b in *E. coli* (Fig. 4). Bands with apparent molecular weight of 32 kDa (Fos13CrtC), 38 kDa (*Rp*CrtC), and 44 kDa (*Rr*CrtC, *Br*CrtC, *Tr*CrtC, and *Rg*CrtC) were visualized on SDS-PAGE and were consistent with the values calculated from the deduced amino acid sequences. *Tr*CrtC shows two protein bands of 44 and 38 kDa as seen before (Hiseni et al. 2011). No expression band could be identified for Fos06CrtC. Although relatively good expression was achieved for most of the CrtCs, only two were active with lycopene as the substrate in the standard enzymatic assay, i.e., *Rg*CrtC and *Tr*CrtC (data not shown).

The amino acid residues that might be important for catalysis (*vide supra*) were substituted by the amino acid alanine in both active enzymes. In addition, truncated (*Tr*CrtC and *Rg*CrtC) and N-terminal point mutants (*Tr*CrtC) were constructed and analyzed to confirm the importance of the N-terminal part of CrtC for the catalytic activity. The activity of the truncated versions was fully retained. Despite the still unknown reason for the truncation, we were able to identify the cleavage site between S57 and S58 by MS analysis. In order to exclude that this truncation only takes place in recombinant expression, the S58 position was modified by substitutions with valine or glutamine (see Electronic Supplementary Materials, including Fig. S2).

**Fig. 5:**
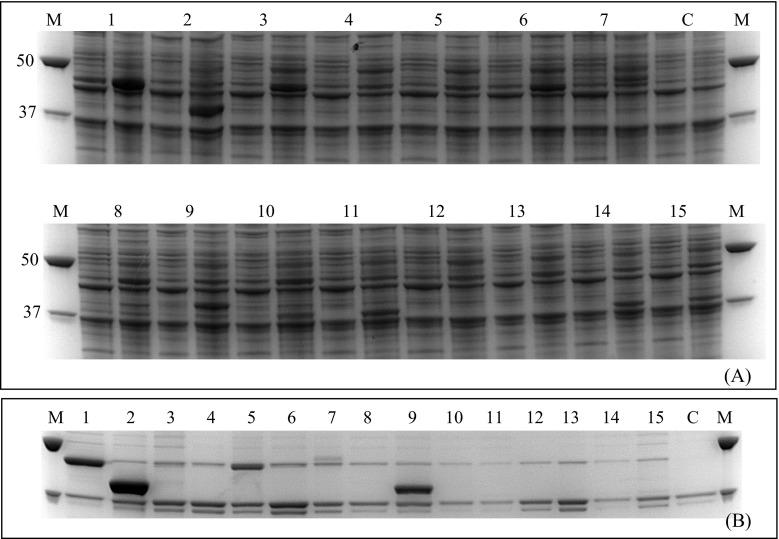
SDS-PAGE (10 %) analysis of expression (**a**) and IMAC purification from membrane (**b**) of CrtCs from *R. gelatinosus* (*Rg*CrtC) (*lanes 1*–*7*) and *T. roseopersicina* (*Tr*CrtC) (*lanes 8*–*15*) wild type and mutants. *M* precision plus protein standard. *C* pET15b control. **a** The *first lane* of each sample shows cells before induction with 0.1 mM IPTG, and the *second lane* shows cells after overnight expression at 25 °C. *1 Rg*CrtC wild type, *2 Rg*CrtC truncated, *3 Rg*CrtC H239A, *4 Rg*CrtC W241A, *5 Rg*CrtC H264A, *6 Rg*CrtC Y266A, *7 Rg*CrtC D268A, *8 Tr*CrtC wild type, *9 Tr*CrtC truncated, *10 Tr*CrtC S58V, *11 Tr*CrtC S58Q, *12 Tr*CrtC H237A, *13 Tr*CrtC W239A, *14 Tr*CrtC H262A, *15 Tr*CrtC D266A

All mutants (Table 2) were successfully cloned and expressed in *E. coli* BL21 (Fig. 5a). However, clear differences in expression levels were observed. While the removal of the N-terminus resulted in an increased expression level, all point mutations negatively influenced the expression of the protein. In order to ensure that CrtC was present, all mutants were purified from the membrane fraction. As can be seen in Fig. 5b, all mutants could be purified by chromatography over a Ni column binding the His tag and showed a band at 38 or 44 kDa, which was absent in the negative control sample (pET15b). In the case of *Tr*CrtC constructs, only very weak protein bands are detected after the purification (Fig. 5b, lanes 8 and 10–15). The purification usually has to be performed as soon as it is expressed (before the cleavage of the N-terminal part including the His tag), which was not the case here.

**Fig. 6:**
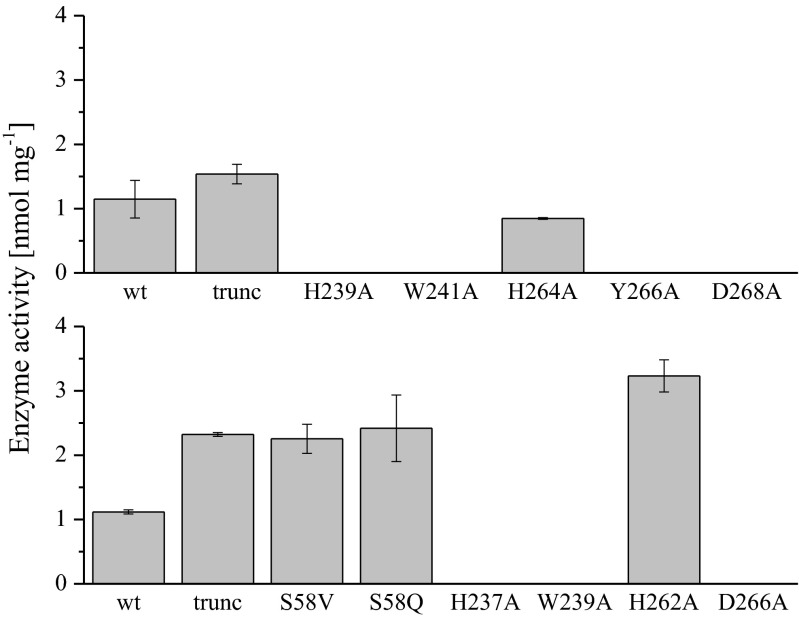
Enzymatic activity of wild type (*wt*) and mutant CrtC from *R. gelatinosus* (*upper*) and *T. roseopersicina* (*lower*). Extracts from *E. coli* cells expressing the respective enzymes were assayed with 20 μM lycopene in 50 mM Na_2_HPO_4_ sodium phosphate (pH 8.0) at 28 °C overnight. *trunc* variants with missing N-terminal residues 1–45 (*Rg*CrtC) and 1–57 (*Tr*CrtC)

Next to the analysis of the expression levels, the activities of all constructed mutants were measured with lycopene as the substrate (Fig. 6). As the expression and purification levels were very low for some of the mutants and the activity of CrtC, in general, is very low, crude extracts were used for the activity assays. Consequently, the results cannot be quantitatively compared. However, in combination with the expression levels as shown in Fig. 5a, indicative conclusions can be drawn. When looking at the results from the alanine mutants, it appears that four key residues were identified, which have a potentially important role in the hydration mechanism. By replacing each of the amino acids H239, W241, Y266, and D268 individually by an alanine in *Rg*CrtC, the activity is completely destroyed. The same mutations of the corresponding amino acids in *Tr*CrtC, i.e., H237, W239, and D266, also resulted in CrtC inactivation. Unfortunately, the mutagenesis of Y264 in *Tr*CrtC was not successful and, therefore, could not be included in this study. However, based on all the results, one could expect that the mutation of Y264 in *Tr*CrtC would lead to inactivation, as seen in *Rg*CrtC. On the other hand, the less conserved H264 in *Rg*CrtC and the corresponding histidine residue in *Tr*CrtC (H262) seem not to have any functional role. The mutants fully retained activity and even showed slightly increased activity when the expression levels were considered. For instance, the truncated *Tr*CrtC and H262A mutant showed almost the same level of expression (Fig. 5a, lanes 9 and 14) but the activity of H262A mutant was ~1.3-fold higher (Fig. 6). The same was observed for *Rg*CrtC, where the expression of the wild type is much higher than that of the mutant H264A, but both showed approximately the same activity.

## Discussion

The main purpose of this study was to get more insight into the hydration mechanism of carotenoid 1,2-hydratases. First, the distribution of these enzymes was assessed by subjecting the *Rg-crtC* nucleotide sequence to a BLAST search. Although *R. gelatinosus* belongs to the Betaproteobacteria, more than 69 % of the identified 119 hits were from Alphaproteobacteria and only 11 % from Betaproteobacteria. Similarly, Igarashi et al. (2001) observed that most of the photosynthesis gene products from *R. gelatinosus* showed high sequence identities to the gene products of *R. palustris*, an Alphaproteobacteria member. They explain this occurrence as horizontal transfer of the photosynthesis gene clusters from an ancestral species belonging to the Alphaproteobacteria to that of the Betaproteobacteria, which might also have happened to the CrtC genes. The identified CrtC sequences were aligned in order to discover conserved group clusters. In total, 33 amino acids were found to be fully conserved. No conserved residues were identified in the N-terminal part of the sequence (amino acids 1–125 in *Rg* numbering), which is in agreement with our earlier hypothesis that this region is probably not necessary for CrtC activity (Hiseni et al. 2011). This was also confirmed by the activity of the truncated variants. Furthermore, this also accounts for the absence of this part and, thus, the shorter DNA sequences for many CrtCs, including Fos06, Fos13, and partly *Rp*CrtC, when compared to *Rg*CrtC (Fig. 2, Supplemental Fig. S1). This part is, hence, not involved in the catalytic mechanism.

Six out of the seven selected CrtCs could be overexpressed in *E. coli* (Fig. 4). The fact that all CrtCs share highly conserved regions in the amino acid sequence indicates that they are performing the same or similar biochemistry. However, no activity whatsoever could be detected for four out of six CrtCs in the standard lycopene hydration assay. At this point, it is unclear whether this is due to reasons of low activity in the cell extract and/or substrate specificity. Since CrtCs are active in different parts of the carotenoid pathway, substrate specificities can differ depending on the carotenoid produced by the organism where the enzyme originated (Kovács et al. 2003).

We have stated earlier that the hydration can take place through an acid–base-type catalysis, resulting in the identification of five possible key residues. The identified key residues H239, W241, Y266, and D268 in *Rg*CrtC and the corresponding residues in *Tr*CrtC were probed by generating alanine point mutants thereof. The absence of activity upon individual substitution leads to the hypothesis that they are involved in the hydration catalysis. Furthermore, they are all in close distance to each other on the protein chain (Fig. 2). The fact that there is only one other residue between H239 and W241 or Y266 and D268 is consistent with residues in space pointing in the same direction in a beta strand or loop (one residue pointing upward, the next downward). In order to investigate how these newly identified key residues could be involved in the catalytic hydration reaction, we built a 3D structure of *Tr*CrtC by homology modeling. The closest known 3D structure, the putative AttH protein from *Nitrosomonas europaea*, showed only a sequence identity of 17 % to the CrtC (see Electronic Supplementary Material Figs. S3, S4, and S5). This is not enough for a reliable model. However, this model does indicate the possibility of the presence of such an active site.

**Fig. 7:**
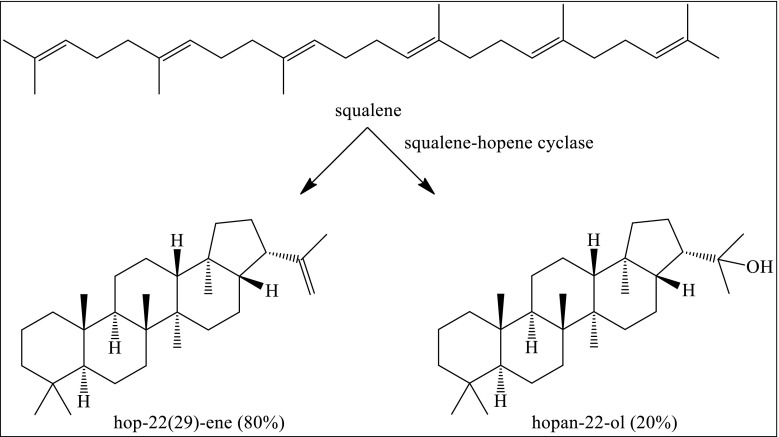
Enzyme-catalyzed cyclisation of squalene to hopene and hopanol

These four residues, which are conserved throughout the whole CrtC family, are also found in the active site of squalene–hopene cyclase (SHC) (Wendt et al. 2000). SHC catalyzes the cyclization reaction of squalene to hopene as a major product (Fig. 7). Hopanol is also formed to a minor extent. The proposed mechanism for cyclases is proton-triggered polycyclization, whereby the intermediate carbocation is stabilized by aromatic amino acids. Next to the stabilization role of the aromatic amino acids, they also create a hydrophobic environment in order to prevent quenching of the cation by water. The cyclization cascade is terminated by a well-positioned enzymatic base. The formation of the alcohol side product suggests significant water accessibility at the termination region of the active site. The acidic residue aspartate (D376), which is located in the center of the active site in SHC, is the likely general acid responsible for protonating the C3 atom of the squalene substrate (Wendt et al. 2000). The acidity of D376 is enhanced by a connection to the side chain of Y495 through a water molecule. The tyrosine positions the proton on aspartic acid into the anti-orientation, turning it more acidic. Carboxylic acid protons in anti-orientation have been estimated to be 10^4^ times more acidic than the biologically more relevant syn-oriented protons (Gandour 1981).

**Fig. 8:**
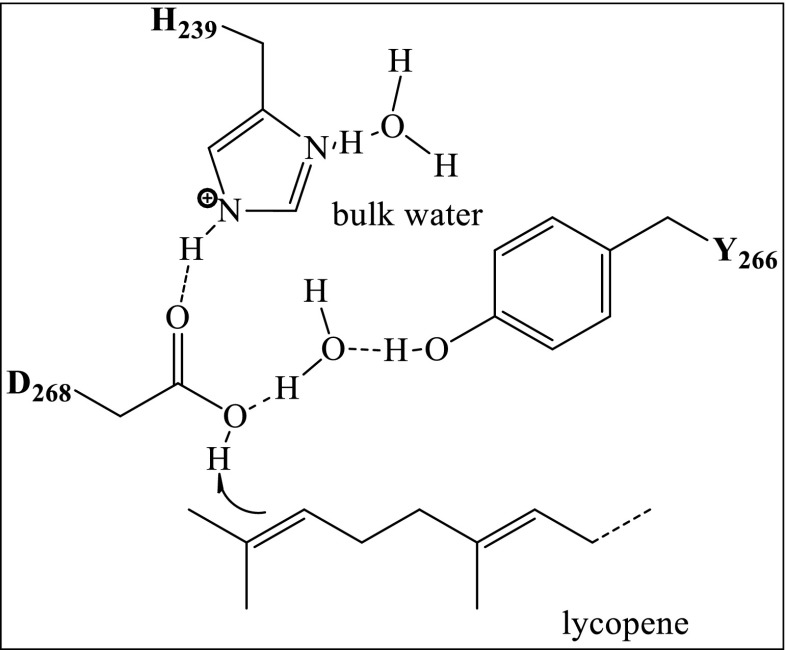
Proposed mechanism for the initial protonation during lycopene hydration

Because of the probable similarity of the initial protonation reactions of squalene and lycopene, we assume that the residues involved in catalysis will be alike in SHC and CrtC. Therefore, we propose the following mechanism for *Rg*CrtC. D268 is the catalytic acid that initiates the hydration of lycopene (Fig. 8). Upon diffusion of lycopene into the active site, the C2 atom of the substrate needs to be positioned near the proton of D268 that putatively will be added onto the substrate. In order to enhance the acidity of the catalytic D268 for olefin protonation, the amino acid is directly bonded to H239 and to Y266 through an ordered water molecule, similar to what has been proposed for SHC (Wendt et al. 2000). This hypothesis is supported by the results that mutation of one of these three amino acids leads to inactivation of enzymatic activity. In contrast to SHC, where premature quenching of the cationic intermediate by water or nucleophiles is prevented by well-positioned aromatic amino acids, a water molecule is added to lycopene to yield the desired hydroxylated lycopene derivative. This suggests that the active site of CrtC has more water molecules present, so that the interaction between the substrate and solvent water molecules is more significant. The aromatic amino acid Trp266 might be involved in the correct positioning of the hydrophobic substrate and the stabilization of the intermediate carbocation.

In conclusion, overall results indicate that residues H239, W241, Y266, and D268 in *Rg*CrtC are probably involved in an acid–base-type hydration. The absence of activity upon individual substitution of these residues by an alanine agrees with a role in the catalytic cycle. We hypothesize that they are involved in the initial protonation, which would be followed by quenching of the carbocation by a water molecule, resulting in the hydration product. From our findings, it becomes clear that the complete structure of a CrtC, through crystallization studies, will be pivotal to really unravel the mechanism for this intriguing enzyme. Nevertheless, the results of this study produce for the first time a workable hypothesis for the catalytic mechanism of carotenoid 1,2-hydratase and open the field for the engineering of this enzyme towards industrially relevant mutants.

## Electronic supplementary material

ESM 1 (PDF 582 kb)
