# Supplementary material for: Identification of catalytically important residues of the carotenoid 1,2-hydratases from Rubrivivax gelatinosus and Thiocapsa roseopersicina
Source: Appl Microbiol Biotechnol. 2015 Oct 19;100:1275–84. doi: 10.1007/s00253-015-6998-y (PMC4717167; doi:10.1007/s00253-015-6998-y)
Supplement: Supplementary file 1 — (PDF 582 kb) [file 253_2015_6998_MOESM1_ESM.pdf]

Electronic Supplementary Material for:

Applied Microbiology and Biotechnology

Identification of catalytically important residues of the carotenoid 1,2-hydratases from *Rubrivivax gelatinosus* and *Thiocapsa roseopersicina*

Aida Hiseni, Linda G. Otten and Isabel W.C.E. Arends*

Biocatalysis Group, Department of Biotechnology, Delft University of Technology, Delft, NL

* Corresponding author: Prof. Dr. Isabel W.C.E. Arends, Biocatalysis Group, Department of Biotechnology, Delft University of Technology, Julianalaan 67, 2628 BC Delft, The Netherlands. Tel.: +31 15 2786659; Fax: +31 15 2781415; E-mail: I.W.C.E.Arends@tudelft.nl;
URL: www.bt.tudelft.nl/boc.

Contents

1. Importance of the N-terminal part of CrtC **p.24**
2. Homology model of CrtC **p.26**

Table S1 **p.24**
Figure S1 **p.2**
Figure S2 **p.25**
Figure S3 **p.26**
Figure S4 **p.27**
Figure S5 **p.28**

Figure S1. Multiple sequence alignment of all CrtC protein sequences showing conserved amino acids. Identical amino acids are marked with an asterisk. *Rg*CrtC sequence is highlighted in yellow (next page).

```
CLUSTAL 2.1 multiple sequence alignment


gi|350284778|gb|AEQ27763.1|_1-      MRGEYSQTAGTLRPEPGVAAGERGRHYTDAVQPDVPGDGGLPVRAGLTRM 50
gi|521992738|ref|WP_020504009.      --------------------------------------------------
gi|557030915|gb|ESQ08988.1|_1-      --------------------------------------------------
gi|494353717|ref|WP_007191968.      --------------------------------------------------
gi|570733543|gb|AHF04706.1|_24      --------------------------------------------------
gi|760081160|ref|WP_043763735.      --------------------------------------------------
gi|528190751|gb|EPY01080.1|_43      --------------------------------------------------
gi|748594904|ref|WP_039853084.      --------------------------------------------------
gi|488815067|ref|WP_002727473.      ------------------LRPSGKMRPSHQAAGGDRLAAMDGLDSYPSDG 32
gi|505094729|ref|WP_015281831.      --------------------------------------------------
gi|754165669|ref|WP_041786299.      --------------------------------------------------
gi|739596279|ref|WP_037453842.      --------------------------------------------------
gi|764363842|ref|WP_044336870.      --------------------------------------------------
gi|644239143|ref|WP_025293539.      --------------------------------------------------
gi|503035479|ref|WP_013270455.      --------------------------------------------------
gi|617472745|gb|KAI93218.1|_2-      ---------------------------AAGFRDASAGNGRRDLRPGLARL 23
gi|737603811|ref|WP_035574467.      --------------------------------------------------
gi|648530966|ref|WP_026222717.      --------------------------------------------------
gi|288897641|gb|ADC63477.1|_28      ---------------------------ARGVRTALSGHGRRALWSGDPRL 23
gi|760114352|ref|WP_043796307.      --------------------------------------------------
gi|494098690|ref|WP_007039487.      --------------------------------------------------
gi|75388571|sp|Q7X3G5.1|CRTC_T      --------------------------DAGRLQPALPGDGRGPLRPGVTRM 24
gi|390426908|gb|AFL73973.1|_35      --------------------------------------------------
gi|752807498|ref|WP_041446922.      --------------------------------------------------
gi|760074757|ref|WP_043757486.      --------------------------------------------------
gi|918211006|ref|WP_052348303.      --------------------------------------------------
gi|499982040|ref|WP_011662758.      --------------------------------------------------
gi|493840365|ref|WP_006787509.      --------------------------------------------------
gi|653037601|ref|WP_027289289.      --------------------------------------------------
gi|519009821|ref|WP_020165696.      --------------------------------------------------
gi|518759539|ref|WP_019916969.      --------------------------------------------------
gi|495340016|ref|WP_008064750.      --------------------------------------------------
gi|517223140|ref|WP_018411958.      --------------------------------------------------
gi|889919304|ref|WP_048861895.      --------------------------------------------------
gi|496441596|ref|WP_009150441.      -----------------------PPGLTDPVRAAVPDQRWRHLWPRIPRL 27
gi|146409846|gb|ABQ38352.1|_10      ---------AALRPQRRDDTGTDPRDDAGGFQPDVSGHGGRAVRPQLAWL 41
gi|908659074|ref|WP_049795525.      --------------------------------------------------
gi|365289544|emb|CCD93979.1|_1      ---------AALRPEHRDDTRIDPRDDAGGFQPDVSGHGGRAVRPQLARL 41
gi|41018902|gb|AAR98493.1|_10-      ---------AALRPEHRDDAGIDPRDDAGGFQPDVSGHGGRVVRPQLARL 41
gi|653027709|ref|WP_027279528.      --------------------------------------------------
gi|499791021|ref|WP_011471755.      --------------------------------------------------
```

```
gi|499823412|ref|WP_011504146.      --------------------------------------------------
gi|499762149|ref|WP_011442883.      --------------------------------------------------
gi|829076993|ref|WP_047310044.      --------------------------------------------------
gi|503269006|ref|WP_013503667.      --------------------------------------------------
gi|499470438|ref|WP_011157078.      --------------------------------------------------
gi|501486651|ref|WP_012495141.      --------------------------------------------------
gi|550975051|ref|WP_022723265.      --------------------------------------------------
gi|764538658|ref|WP_044412067.      --------------------------------------------------
gi|754113006|ref|WP_041748751.      --------------------------------------------------
gi|737680904|ref|WP_035649991.      --------------------------------------------------
gi|503405597|ref|WP_013640258.      --------------------------------------------------
gi|657196689|ref|WP_029312388.      --------------------------------------------------
gi|703385326|ref|WP_033417843.      --------------------------------------------------
gi|653032131|ref|WP_027283892.      --------------------------------------------------
gi|378403832|emb|CCG08948.1|_1      -------------PGPRPTG-----DDTARLQHDVSRHGGSPVRPLDPRL 32
gi|754176764|ref|WP_041795395.      --------------------------------------------------
gi|25989732|gb|AAN75036.1|_12-      -----------LRPNAAPDAGGDPGNDSGRLQQPVPGDGGSALRPGVARV 39
gi|860504149|ref|WP_048445338.      --------------------------------------------------
gi|860474721|ref|WP_048434413.      --------------------------------------------------
gi|501291381|ref|WP_012333472.      --------------------------------------------------
gi|860453591|ref|WP_048427078.      --------------------------------------------------
gi|860560381|ref|WP_048464709.      --------------------------------------------------
gi|860527577|ref|WP_048454269.      --------------------------------------------------
gi|760871815|dbj|BAQ48812.1|_1      --------------------------------------------------
gi|373567336|gb|EHP93308.1|_6-      --------------------------------------------------
gi|240009448|gb|ACS40674.1|_4-      --------------------------------------------------
gi|218523206|gb|ACK83791.1|_4-      --------------------------------------------------
gi|254269168|emb|CAX25134.1|_4      --------------------------------------------------
gi|163663749|gb|ABY31116.1|_2-      --------------------------------------------------
gi|494837541|ref|WP_007563641.      --------------------------------------------------
gi|914802769|ref|WP_050733395.      --------------------------------------------------
gi|657246165|ref|WP_029357447.      --------------------------------------------------
gi|501276758|ref|WP_012319776.      --------------------------------------------------
gi|640229117|ref|WP_024829694.      --------------------------------------------------
gi|518936109|ref|WP_020091984.      --------------------------------------------------
gi|760075276|ref|WP_043757989.      --------------------------------------------------
gi|759662308|ref|WP_043379914.      --------------------------------------------------
gi|498368472|ref|WP_010682628.      --------------------------------------------------
gi|497224411|ref|WP_009538673.      --------------------------------------------------
gi|589291345|gb|AHK79488.1|_12      --------------------------------------------------
gi|644125233|ref|WP_025281955.      --------------------------------------------------
gi|496218797|ref|WP_008932834.      --------------------------------------------------
gi|495659402|ref|WP_008383981.      --------------------------------------------------
gi|687960265|gb|KGE05197.1|_16      --------------------------------------------------
gi|496312569|ref|WP_009021747.      --------------------------------------------------
gi|495571757|ref|WP_008296336.      --------------------------------------------------
```

```
gi|908380581|ref|WP_049751705.      --------------------------------------------------
gi|658524263|ref|WP_029718812.      --------------------------------------------------
gi|332109922|gb|EGJ10550.1|_1-      --------------------------------------------------
gi|760103377|ref|WP_043785587.      --------------------------------------------------
gi|760129436|ref|WP_043810804.      --------------------------------------------------
gi|7416798|dbj|BAA94047.1|_1-4      MRAAESGADARVRPDDRLEPADAPAGDAGELRAPVPGDGGSAVRPGDARL 50
gi|75345570|sp|P95619.1|CRTC_R      MRAAESGADARVRPVDRVEPADAPAGDAGGLRAAVPGDGGSAVRPGDARL 50
gi|913634290|gb|KNZ32951.1|_1-      --------------------------------------------------
gi|809277751|ref|WP_046331236.      --------------------------------------------------
gi|518258896|ref|WP_019429104.      --------------------------------------------------
gi|518255752|ref|WP_019425960.      --------------------------------------------------
gi|918152310|gb|KOF08324.1|_1-      --------------------------------------------------
gi|61653243|gb|AAX48196.1|_1-2      --------------------------------------------------


gi|350284778|gb|AEQ27763.1|_1-      AGAIQPVGGAQPGAGALSGGGERASGSGDADGGDLRSLGRGGRAAGPDFG 100
gi|521992738|ref|WP_020504009.      --------------------------------------------------
gi|557030915|gb|ESQ08988.1|_1-      --------------------------------------------------
gi|494353717|ref|WP_007191968.      --------------------------------------------------
gi|570733543|gb|AHF04706.1|_24      ---------------------------------------------APDFN 5
gi|760081160|ref|WP_043763735.      ---------------------------------------------APDFN 5
gi|528190751|gb|EPY01080.1|_43      -----LIRPSELGSPDPGPLPCGRRFVQAGPAQTATGPLGLGGDRGPRFD 45
gi|748594904|ref|WP_039853084.      --------------------------------------------------
gi|488815067|ref|WP_002727473.      KIGGGMIRPSELGSSDPGPLPCGRSFVQ-GSGQTGNVPLGVAGDRGPRFD 81
gi|505094729|ref|WP_015281831.      --------------------------------------------------
gi|754165669|ref|WP_041786299.      ------------------------------------------------FD 2
gi|739596279|ref|WP_037453842.      --------------------------------------------------
gi|764363842|ref|WP_044336870.      ----------------------------------------------PRFD 4
gi|644239143|ref|WP_025293539.      ----------------------------------------------PRFD 4
gi|503035479|ref|WP_013270455.      --------------------------------------------------
gi|617472745|gb|KAI93218.1|_2-      DGVVPETGDALDHAGPLFRGGERSSGAGHSDGGAFWRSRIGGASCGPRFA 73
gi|737603811|ref|WP_035574467.      --------------------------------------------------
gi|648530966|ref|WP_026222717.      --------------------------------------------------
gi|288897641|gb|ADC63477.1|_28      EGVVLASSLAHQDSGTLSGGREHASGRGHSHGGDVGPTGGGSATRGPAFD 73
gi|760114352|ref|WP_043796307.      --------------------------------------------------
gi|494098690|ref|WP_007039487.      --------------------------------------------------
gi|75388571|sp|Q7X3G5.1|CRTC_T      EGLLHASSVAQQGAGALSGRGERASGSRGTDGGHVGTPGGSDPARGPRFD 74
gi|390426908|gb|AFL73973.1|_35      ---------------ASPQGAGRAPGAEGIHGDELALAGGCEPGCGPRFD 35
gi|752807498|ref|WP_041446922.      --------------------------------------------------
gi|760074757|ref|WP_043757486.      ----------------------------------------------PRFD 4
gi|918211006|ref|WP_052348303.      --------------------------------------------------
gi|499982040|ref|WP_011662758.      ----------------------------------------------PRFD 4
gi|493840365|ref|WP_006787509.      --------------------------------------------------
gi|653037601|ref|WP_027289289.      ------------------------------------------NTDGPGFD 8
gi|519009821|ref|WP_020165696.      --------------------------------------------NARNFD 6
gi|518759539|ref|WP_019916969.      ------------------------------------------------FN 2
```

```
gi|495340016|ref|WP_008064750.      ------------------------------------------------FS 2
gi|517223140|ref|WP_018411958.      ---------------------------------------------ARRFD 5
gi|889919304|ref|WP_048861895.      ------------------------------------------------FS 2
gi|496441596|ref|WP_009150441.      DGVFQSTGSTHQAAGPVSGGGRPPPGTGTAEGDPIRPSDSADRARGPAFD 77
gi|146409846|gb|ABQ38352.1|_10      DGVVPAPGGADETAGTLHSRGQRASGAGRADGGAVGPDGSRAGDSGPRFD 91
gi|908659074|ref|WP_049795525.      --------------------------------------------------
gi|365289544|emb|CCD93979.1|_1      DGVVPAAGCADEAGRALRGGGQRASGTGRADGSAVGPDGSRTGDSGPRFD 91
gi|41018902|gb|AAR98493.1|_10-      DGIIPAAGRADEAARALRGGGQRPSGTGRPDGGLVGPDGRRQGDLRPRFD 91
gi|653027709|ref|WP_027279528.      ------------------------------------------------FN 2
gi|499791021|ref|WP_011471755.      ------------------------------------------------FN 2
gi|499823412|ref|WP_011504146.      ------------------------------------------------FN 2
gi|499762149|ref|WP_011442883.      ------------------------------------------------FN 2
gi|829076993|ref|WP_047310044.      ------------------------------------------------FN 2
gi|503269006|ref|WP_013503667.      ------------------------------------------------FN 2
gi|499470438|ref|WP_011157078.      ------------------------------------------------FN 2
gi|501486651|ref|WP_012495141.      ------------------------------------------------FN 2
gi|550975051|ref|WP_022723265.      ------------------------------------------------FN 2
gi|764538658|ref|WP_044412067.      ------------------------------------------------FN 2
gi|754113006|ref|WP_041748751.      -------------------------------------MGGSQPARGPDFS 13
gi|737680904|ref|WP_035649991.      -------------------------------------------DRGPDFS 7
gi|503405597|ref|WP_013640258.      -----------------------------------------------RFD 3
gi|657196689|ref|WP_029312388.      -----------------------------------------------RFD 3
gi|703385326|ref|WP_033417843.      --------------------------------------------------
gi|653032131|ref|WP_027283892.      ------------------------------------------------FD 2
gi|378403832|emb|CCG08948.1|_1      ERLLRSDGERDGAAGPVGRGGRGPSGAGDSDGGALGPAGGTGGPAGPDLM 82
gi|754176764|ref|WP_041795395.      --------------------------------------------------
gi|25989732|gb|AAN75036.1|_12-      VGDLRPAGGENGDARPLSGGGQRPSGTGGADGGLIGTVGGPGGSGGPDFA 89
gi|860504149|ref|WP_048445338.      --------------------------------------------------
gi|860474721|ref|WP_048434413.      --------------------------------------------------
gi|501291381|ref|WP_012333472.      --------------------------------------------------
gi|860453591|ref|WP_048427078.      --------------------------------------------------
gi|860560381|ref|WP_048464709.      --------------------------------------------------
gi|860527577|ref|WP_048454269.      --------------------------------------------------
gi|760871815|dbj|BAQ48812.1|_1      --------------------------------------------------
gi|373567336|gb|EHP93308.1|_6-      ----------------------------------------------PRFD 4
gi|240009448|gb|ACS40674.1|_4-      --------------------------------------------------
gi|218523206|gb|ACK83791.1|_4-      --------------------------------------------------
gi|254269168|emb|CAX25134.1|_4      --------------------------------------------------
gi|163663749|gb|ABY31116.1|_2-      --------------------------------------------------
gi|494837541|ref|WP_007563641.      --------------------------------------------------
gi|914802769|ref|WP_050733395.      --------------------------------------------------
gi|657246165|ref|WP_029357447.      --------------------------------------------------
gi|501276758|ref|WP_012319776.      --------------------------------------------------
gi|640229117|ref|WP_024829694.      --------------------------------------------------
gi|518936109|ref|WP_020091984.      --------------------------------------------------
gi|760075276|ref|WP_043757989.      --------------------------------------------------
```

```
gi|759662308|ref|WP_043379914.      --------------------------------------------------
gi|498368472|ref|WP_010682628.      --------------------------------------------------
gi|497224411|ref|WP_009538673.      --------------------------------------------------
gi|589291345|gb|AHK79488.1|_12      ----------QPDTGAVPDRGERASGRGRADGEPLGTSGSIGPASGPDFT 40
gi|644125233|ref|WP_025281955.      --------------------------------------------------
gi|496218797|ref|WP_008932834.      --------------------------------------------------
gi|495659402|ref|WP_008383981.      --------------------------------------------------
gi|687960265|gb|KGE05197.1|_16      --------------------------------------------------
gi|496312569|ref|WP_009021747.      ------------------------SPKNQREANADLPAGYNSRYPGLNFA 26
gi|495571757|ref|WP_008296336.      ------------------------------------------------FA 2
gi|908380581|ref|WP_049751705.      --------------------------------------------------
gi|658524263|ref|WP_029718812.      --------------------------------------------------
gi|332109922|gb|EGJ10550.1|_1-      --------------------------------------------------
gi|760103377|ref|WP_043785587.      --------------------------------------------------
gi|760129436|ref|WP_043810804.      --------------------------------------------------
gi|7416798|dbj|BAA94047.1|_1-4      DVLVPPGLVDEPAAGPVPGGRQRAPGAGRADGGDVRPAGGRDADGAPRFD 100
gi|75345570|sp|P95619.1|CRTC_R      DVLVPPGLVDEPAAGALPGGGQRAPGAGRADGGDVRPVGGRDADGAPRFD 100
gi|913634290|gb|KNZ32951.1|_1-      --------------------------------------------------
gi|809277751|ref|WP_046331236.      --------------------------------------------------
gi|518258896|ref|WP_019429104.      --------------------------------------------------
gi|518255752|ref|WP_019425960.      --------------------------------------------------
gi|918152310|gb|KOF08324.1|_1-      --------------------------------------------------
gi|61653243|gb|AAX48196.1|_1-2      --------------------------------------------------

gi|350284778|gb|AEQ27763.1|_1-      VSVGPRGYAWWYVDALSDDGRHGLTLIAFVGSVFSPYYFRGRRRGL---- 146
gi|521992738|ref|WP_020504009.      --VGPRGYAWWYVDALSDDGRHGLTLIAFVGSVFSPYYFRGRRRGL---- 44
gi|557030915|gb|ESQ08988.1|_1-      ------------MDALSDDGKHGLTLIAFIGSVFSPYYFKGRRRNK---- 34
gi|494353717|ref|WP_007191968.      ------------MDALSDDGRHGLTLIAFIGSVFSPYYFKGRRRGR---- 34
gi|570733543|gb|AHF04706.1|_24      IDVPPRGYAWWYVDALSDDGRHGLTIIAFIGSVFSPYYFKGRHRGK---- 51
gi|760081160|ref|WP_043763735.      IDVPPRGYAWWYVDALSDDGRHGLTIIAFIGSVFSPYYFKGRHRGK---- 51
gi|528190751|gb|EPY01080.1|_43      RTIPGSGYSWWYIDALSDDGKHGLTLIFFIGSVFSPYYAWARRKGG---T 92
gi|748594904|ref|WP_039853084.      --MPGSGYSWWYIDALSDDGKHGLTLIFFIGSVFSPYYAWARRKGG---T 45
gi|488815067|ref|WP_002727473.      RQIPRSGYSWWYIDALSDDGQHGLTLIFFIGSVFSPYYAWARRRGGG--A 129
gi|505094729|ref|WP_015281831.      ---------------MSDDGRHGLTLIAFIGSVFSPYYAWARRRGR---- 31
gi|754165669|ref|WP_041786299.      RKVPRDGYAWWYVDALSDDGRYGLTLIAFIGSVFSPYYAWARRRGE---- 48
gi|739596279|ref|WP_037453842.      ---------------MSEDGRHGLTVIAFIGSVFSPYYAWARRRGR---- 31
gi|764363842|ref|WP_044336870.      VHVAANGYAWWYIDATSADGAHGLTIIAFIGSVFSPYYKLSGR------- 47
gi|644239143|ref|WP_025293539.      VAVAPNGYAWWYIDATSADGAYGLTIIAFIGSVFSPYYKLSGR------- 47
gi|503035479|ref|WP_013270455.      ---------------MSADGRHGLTIIAFVGSVFSPWYAWSGR------- 28
gi|617472745|gb|KAI93218.1|_2-      ETVEPNGYAWWYVDAFSADHRYGLTIIAFIGSVFSPYYAKERRRAI---- 119
gi|737603811|ref|WP_035574467.      ---------------MSDDGRHGLTIIAFVGSVFSPYYAWARARRT---- 31
gi|648530966|ref|WP_026222717.      ---------------MSDDGEYGVTVIAFIGSVFSPYYAWSGW------- 28
gi|288897641|gb|ADC63477.1|_28      LTVDNDGYVWWYVDALSDDEQYGLTLIAMLGSVFSPYYAWSRQR----GN 119
gi|760114352|ref|WP_043796307.      ---------------MSDDEQYGLTLIAMLGSVFSPYYAWSRQR----GN 31
gi|494098690|ref|WP_007039487.      ---------------MSDDGQHGLTLIALIGSVFSPYYAWSRQR----GQ 31
```

```
gi|75388571|sp|Q7X3G5.1|CRTC_T      LRITPGGYLWWYLDALSDDGDHGLTIIAMLGSVFSPYYAWARRR----GN 120
gi|390426908|gb|AFL73973.1|_35      APVMPEGYLWWYVDAFSDDRRHGITLIAQVGSCFSPYYAWARRR----GD 81
gi|752807498|ref|WP_041446922.      ----PEGYLWWYVDAFSDDRRHGITLIAQVGSCFSPYYAWARRR----GD 42
gi|760074757|ref|WP_043757486.      AGVMPEGYLWWYVDALSDDGRYGITLIAQLGSCFSPYYAKARRR----GD 50
gi|918211006|ref|WP_052348303.      ----PEGYLWWYVDALSDDGRYGITLIAQLGSCFSPYYAKARRR----GD 42
gi|499982040|ref|WP_011662758.      APVVRGGYSWWYVDALSDDGKNGITLIALIGSVFSPYYAAMRRRP---IG 51
gi|493840365|ref|WP_006787509.      ------------MDAFSEDHRFGIILIAMLGSVFSPYYARSRAR----GE 34
gi|653037601|ref|WP_027289289.      QPVAPGGYVWWYVDALSDDGQHGLTIIAFIGSVFSPYYAWARQRAP-GGA 57
gi|519009821|ref|WP_020165696.      AAVAPGGYRWWYVDATSDDGRCGLTVIAFIGSVFSPYYALARRRRPG--A 54
gi|518759539|ref|WP_019916969.      APVPAGGYRWWYVDATSDDGRCGLTVIAFIGSVFSPYYALARRRRPG--A 50
gi|495340016|ref|WP_008064750.      APVPPGGYRWWYVDATSDDGRYGLTVIAFIGSVFSPYYALARRRRPG--A 50
gi|517223140|ref|WP_018411958.      TEVPAGGYRWWYIDANSDDGRYGLVIIAFIGSVFSPYYARARRREP---T 52
gi|889919304|ref|WP_048861895.      APVPAGGYAWWYVDAVSDDGRSGLTVIAFVGSVFSPYYSFARRRGP---- 48
gi|496441596|ref|WP_009150441.      QSIPKNGYLWWYIDALSDDGQHGLTIIALLGSVFSPYYARARQRGP---- 123
gi|146409846|gb|ABQ38352.1|_10      RAIPKDGYAWWYVDALSDDGRFGITLIAFIGSVFSPYYAWAR----RRGP 137
gi|908659074|ref|WP_049795525.      --MPKDGYAWWYVDALSDDGRFGITLIAFIGSVFSPYYAWAR----RRGP 44
gi|365289544|emb|CCD93979.1|_1      RTVPTNGYAWWYVDALSDDGRFGLTIIAFVGSVFSPYYAWAR----RRGP 137
gi|41018902|gb|AAR98493.1|_10-      RAVPKDGYAWWYVDALSDDGRFGITVIAFVGSVFSPYYAWAR----RRGP 137
gi|653027709|ref|WP_027279528.      LHVPDNGYAWWYIDAMSDDGTEGLTIITFVGSVFSPYYRFAR----RNQP 48
gi|499791021|ref|WP_011471755.      LPVPDDGYAWWYIDALSDDGQEGLTIIAFVGSVFSPYYRFAR----RRGP 48
gi|499823412|ref|WP_011504146.      LDVPPNGYAWWYVDAISDDGSEGLTIIAFIGSVFSPYYAFAR----RGAA 48
gi|499762149|ref|WP_011442883.      LDVPPNGYAWWYVDAISDDGSEGLTIIAFIGSVFSPYYKFAR----RDAP 48
gi|829076993|ref|WP_047310044.      VDIPRNGYAWWYIDAISDDGREGLTIIAFIGSVFSPYYAFAR----RRGP 48
gi|503269006|ref|WP_013503667.      VDIPRNGYAWWYIDAISDDGREGLTIIAFIGSVFSPYYAFAR----RRGP 48
gi|499470438|ref|WP_011157078.      VDIPHNGYAWWYIDAISDDGREGLTIIAFIGSVFSPYYAFAR----RRGP 48
gi|501486651|ref|WP_012495141.      VDIPHNGYAWWYIDAISDDGREGLTIIAFIGSVFSPYYAFAR----RRAP 48
gi|550975051|ref|WP_022723265.      LDVPRNGYAWWYVDAISDDGREGLTIIAFIGSVFSPYYAFAR----RRAP 48
gi|764538658|ref|WP_044412067.      LDVPRNGYAWWYIDALSDDGREGLTIIAFVGSVFSPYYAFAR----RKGA 48
gi|754113006|ref|WP_041748751.      RIVPRNGYAWWYVDALSDDGQNGITIIAFIGSVFSPYYAFAR----RKGP 59
gi|737680904|ref|WP_035649991.      RVVPPNGYAWWYIDALSDDGHNGITIIAFIGNVFSPYYAFAR----RKRP 53
gi|503405597|ref|WP_013640258.      RPVPRGGYAWWYVDALSDDGRHALTLIAFIGSVFSPYYAAARRL--APGR 51
gi|657196689|ref|WP_029312388.      HPIPRGGYAWWYVDALSDDGLHALTLIAFIGSVFSPYYAAANRR--TPGG 51
gi|703385326|ref|WP_033417843.      -----GGYRWWYLDGLSDDGEHALTLIAFIGSVFSPYYKRAR----KHGS 41
gi|653032131|ref|WP_027283892.      RPVPERGYAWWYVDALSEDGTQGITLIAFIGTVFSPWYALAR----RGGG 48
gi|378403832|emb|CCG08948.1|_1      RSVKPGGYLWWYVDALSDDGRSGLTLIAFIGSVFSPYYAWAGRK------ 126
gi|754176764|ref|WP_041795395.      --VKPGGYLWWYVDALSDDGRSGLTLIAFIGSVFSPYYAWAGRK------ 42
gi|25989732|gb|AAN75036.1|_12-      RPVAPGGYAWWYVDAFSDDGAQAFTLIAFVGSVFSPYYHWAKRR------ 133
gi|860504149|ref|WP_048445338.      --VCPGGYAWWYVDGVSDDGRHGITVIAFLGSVFSPYYAWSGRA------ 42
gi|860474721|ref|WP_048434413.      --MPAGGYAWWYVDGVSDDGRHGITVIAFLGSVFSPYYAWSRDK------ 42
gi|501291381|ref|WP_012333472.      --MPPGGYAWWYVDGVSDDGRHGLTVIAFLGSVFSPYYAWSGRQ------ 42
gi|860453591|ref|WP_048427078.      ---------------MSDDGQHGLTIIAFLGSVFSPYYAWSGRG------ 29
gi|860560381|ref|WP_048464709.      ---------------MSDDGQHGLTVIAFLGSVFSPYYAWSGRG------ 29
gi|860527577|ref|WP_048454269.      --MPPGGYAWWYVDAVSDDGEHGLTVIAFLGSVFSPYYAWSGRG------ 42
gi|760871815|dbj|BAQ48812.1|_1      ---------------MSDDGQHGLTVIAFLGSVFSPYYAWSGRG------ 29
gi|373567336|gb|EHP93308.1|_6-      GAVTRGGYVWWYVDAVSADGRQGLTVIAFIGSVFSPYYAWDAAR------ 48
gi|240009448|gb|ACS40674.1|_4-      -----GGYVWWYVDAVSADGRQGLTVIAFIGSVFSPYYAWDAAR------ 39
```

```
gi|218523206|gb|ACK83791.1|_4-   -----GGYVWWYVDAVSEDGRQGLTVIAFIGSVFSPYYAWDAAR------ 39
gi|254269168|emb|CAX25134.1|_4   -----GGYVWWYVDAVSEDGRRGLTVIAFIGSVFSPYYAWDAAR------ 39
gi|163663749|gb|ABY31116.1|_2-   --VTRGGYVWWYVDAVSEDGRQGLTVIAFIGSVFSPYYAWDAAR------ 42
gi|494837541|ref|WP_007563641.   ---------------MSDDGRQGLTIIAFIGSVFSPYYGWSADK------ 29
gi|914802769|ref|WP_050733395.   ---------------MSDDGRQGLTIIAFIGSVFSPYYGWSADK------ 29
gi|657246165|ref|WP_029357447.   ---------------MSDDGRQGLTIIAFIGSVFSPYYAWSAEK------ 29
gi|501276758|ref|WP_012319776.   ---------------MSDDGRQGLTIIAFIGSVFSPYYAWSAEK------ 29
gi|640229117|ref|WP_024829694.   ---------------MSDDGRQGLTIIAFIGSVFSPYYAWSAEK------ 29
gi|518936109|ref|WP_020091984.   ---------------MSDDGRQGLTIIAFIGSVFSPYYAWSAEK------ 29
gi|760075276|ref|WP_043757989.   ---------------MSDDGRHGLTIIAFIGSVFSPYYAWSAEK------ 29
gi|759662308|ref|WP_043379914.   ---------------MSDDGRHGLTIIAFIGSVFSPYYAWSAEK------ 29
gi|498368472|ref|WP_010682628.   ---------------MSDDGRQGLTIIAFIGSVFSPYYGWSATK------ 29
gi|497224411|ref|WP_009538673.   ------GYVWWYVDAVSDDGREALTIICFIGSVFSPYYAWTRRKG----P 40
gi|589291345|gb|AHK79488.1|_12   ETVTPGGYLWWYLDALSDDGRYGITLIGFVGSVFSPYYAWARRRGNPD-- 88
gi|644125233|ref|WP_025281955.   ---------------MSDDGRYGITLIGFVGSVFSPYYAWARRRGNPD-- 33
gi|496218797|ref|WP_008932834.   --MTPGGYLWWYLDAWSDDGRHGITLIAFVGSVFSPYYALARRRGTAD-- 46
gi|495659402|ref|WP_008383981.   ---------------MSDDGRHALTIIAFVGSVFSPYYAWARKRAAAPSA 35
gi|687960265|gb|KGE05197.1|_16   --VTAGGYHWWYLDAMSDDGEHALTVIVFVGSVFSPYYAAARRRLAAP-- 46
gi|496312569|ref|WP_009021747.   EPVADDGYLWWYIDAISDDGLQAITMIIFVGSVFSPYYARARRRG----P 72
gi|495571757|ref|WP_008296336.   EAVADNGYLWWYVDAISDDGRQAITMIIFVGSVFSPYYARARRKA----V 48
gi|908380581|ref|WP_049751705.   ---------------MSDDGRHGLTVILFVGSVFSPYYAWARQRG----T 31
gi|658524263|ref|WP_029718812.   -----------------------MTFIAFVGSVFSPYYAWAG-GP-NADR 25
gi|332109922|gb|EGJ10550.1|_1-   --------------------------------------------------
gi|760103377|ref|WP_043785587.   -----------------------MTFIAFVGSVFSPYYAWAG-GP-NADR 25
gi|760129436|ref|WP_043810804.   -----------------------MTFIAFVGSVFSPYYAWAG-GP-NADR 25
gi|7416798|dbj|BAA94047.1|_1-4   QPVPPGGYLWWYVDAVSDDGRHGLTFIAFVGSVFSPYYAWAG-GP-NADR 148
gi|75345570|sp|P95619.1|CRTC_R   QPVPPGGYLWWYVDAVSDDGRHGLTFIAFVGSVFSPYYAWAG-GP-KADR 148
gi|913634290|gb|KNZ32951.1|_1-   -----------------------MTIIAFVGSVFSSYYAWAQRNG-NAGR 26
gi|809277751|ref|WP_046331236.   ---------------MSDDGLYGFSIIAFVGSVFSPYYAWAN----KKQP 31
gi|518258896|ref|WP_019429104.   ---------------MSDDCQHGITLIAFVGSVFSPYYAWAQ----RSGS 31
gi|518255752|ref|WP_019425960.   ---------------MSDDGQHGITLIAFVGSVFSPYYAWAR----RRGR 31
gi|918152310|gb|KOF08324.1|_1-   ---------------MSDCGQHGITLIAFVGSVFSPYYAWAR----KRGA 31
gi|61653243|gb|AAX48196.1|_1-2   ---------------MSDDGQHGITLIAFVGSVFSPYYAWAR----QRGR 31
                                                  *             *   **  *

gi|350284778|gb|AEQ27763.1|_1-   DNPHDYCSLNVCLYGTH-R----RWTMTERRSKAVIQEAAALTIGPSALR 191
gi|521992738|ref|WP_020504009.   DNPHDYCSLNVCLYGTH-R----RWTMTERRSKAVTQEAAALTIGPSALH 89
gi|557030915|gb|ESQ08988.1|_1-   DDPRDYCSLNACLYGQE-R----RWTMTERRAGSLSQSASTLSIGPSNLH 79
gi|494353717|ref|WP_007191968.   DEPSDYCSLNVCLYGKE-R----RWTMTERRRHALAQTASRLQIGPSALQ 79
gi|570733543|gb|AHF04706.1|_24   LDPREYCAVNACLYGEQ-R----RWAMTERGADALTQSDDALTIGPSSVR 96
gi|760081160|ref|WP_043763735.   LDPREYCAVNACLYGEQ-R----RWAMTERGADALTQSDDALTIGPSSVR 96
gi|528190751|gb|EPY01080.1|_43   AEPLDHCSVNVCLYGAT-K----RWSMTERNRSAVNRSIDTLVIGPSALR 137
gi|748594904|ref|WP_039853084.   AEPLDHCSVNVCLYGAT-K----RWSMTERNRSAVNRSIDTLVIGPSALR 90
gi|488815067|ref|WP_002727473.   AEPLDHCSVNVCLYGAAGK----RWSMTERNRNAVHRSTDTLVIGPSALR 175
gi|505094729|ref|WP_015281831.   GEPEHFCSVNVALYGAAGK----RWALTERGRNALSRSTGELTIGPSALQ 77
gi|754165669|ref|WP_041786299.   GDPENFCSLNVVLSGPGAN----RWCMTERGRSSLTAESQGLGIGPSSVR 94
```

```
gi|739596279|ref|WP_037453842.      GDPLNHCTVNVVLTGAGGA----RWCMTERGRGKTRRDASSLTIGPSSLN 77
gi|764363842|ref|WP_044336870.      GDPENHCAINVSLNGPRGS----AWAMTERPSKRMRRSADHFAVGPSGMH 93
gi|644239143|ref|WP_025293539.      QRPENHCAINVSLNGPRGS----AWAMTERPSARVHRSADHFSVGPSGVR 93
gi|503035479|ref|WP_013270455.      GAPENHCALNVAFYGPDCQ----RWTMTERPAGQVEREARHIQIGPSSLS 74
gi|617472745|gb|KAI93218.1|_2-      VDPAQHCSINVAVYGRGAA----RWAMTERCRHALGATPEQLAIGPSSLS 165
gi|737603811|ref|WP_035574467.      VPAENHVALNVALYGPRG-----AWAMTERGAGALAREADRFVVGPSSLT 76
gi|648530966|ref|WP_026222717.      ADPFNHCAVNVALYGPRGA----RWAMTERGRKSLKRTRDVLLIGPSSLE 74
gi|288897641|gb|ADC63477.1|_28      ADPLDHCALNVALYGRRGK----RWAMTERGRAALQRDRTNLVIGPSHVH 165
gi|760114352|ref|WP_043796307.      ADPLDHCALNVALYGRRGK----RWAMTERGRAALQRDRTNLVIGPSHVH 77
gi|494098690|ref|WP_007039487.      ADPLDHCAINVALYGRNGK----RWAMTERGRTSLHRTGDSLTIGPSALA 77
gi|75388571|sp|Q7X3G5.1|CRTC_T      PDPLNHCALNVALYGKAGK----RWTMTERGRKALRQAPGRLDIGPSHLT 166
gi|390426908|gb|AFL73973.1|_35      SDPLNHNSLNVALYG-ACK----RWSMTERGRSALRRDASRLEIGPSALS 126
gi|752807498|ref|WP_041446922.      SDPLNHNSLNVALYG-ACK----RWSMTERGRSALRRDASRLEIGPSALS 87
gi|760074757|ref|WP_043757486.      SDPLNHNSLNVALYG-EVK----RWTMTERGRSALRRDASSLEIGPSALS 95
gi|918211006|ref|WP_052348303.      SDPLNHNSLNVALYG-EVK----RWTMTERGRSALRRDASSLEIGPSALS 87
gi|499982040|ref|WP_011662758.      GDPLHHVSLNVAIYG-EGK----RWSMTERGRGSLQRDKTWLQIGPSSLE 96
gi|493840365|ref|WP_006787509.      LDPLNHCALNVALYGPNGK----RWALTDRGRSSVERGRDFLRIGPSQLC 80
gi|653037601|ref|WP_027289289.      ADPLNHCSINVALYGEGPN----RWAMTERGRTRIDRGARHFSVGPSGLH 103
gi|519009821|ref|WP_020165696.      ADPHAHCAVNVALYGDR-R----RWTMTERGASALQRDTHSLRIGPSRLH 99
gi|518759539|ref|WP_019916969.      ADPHAHCAVNVALYGDK-R----RWTMTERGASALQRDAHSLRIGPSRLH 95
gi|495340016|ref|WP_008064750.      ADPHAHCAVNVALYGDK-R----RWAMTERGASALQRDARNLRIGPSRLH 95
gi|517223140|ref|WP_018411958.      ADPHAHCAVNVALYGDN-R----RWTMTERGPQALQRDAQHLRIGPSHLH 97
gi|889919304|ref|WP_048861895.      VDPERHCAVNVVLYGERGR----RWTMTERGASHLHRTATSFALGPSTLA 94
gi|496441596|ref|WP_009150441.      ADPEHFCALNAVIYGASGK----RWAMTERGRQRLSRTPTELRIGPSQLH 169
gi|146409846|gb|ABQ38352.1|_10      ADPEDHCALNVALYGARAR----RWSMTERGRGAIVRDAESFKIGPSRLA 183
gi|908659074|ref|WP_049795525.      ADPEDHCALNVALYGARAR----RWSMTERGRGAIVRDAESFKIGPSRLA 90
gi|365289544|emb|CCD93979.1|_1      AEPENHCALNVALYGKRAR----RWSMTERGRGAIARDEARFAVGPSSLS 183
gi|41018902|gb|AAR98493.1|_10-      ADAENHCALNVALYGERAR----RWAMTERGRGAIARDEASFAIGPSSLA 183
gi|653027709|ref|WP_027279528.      VDPMNHCAINVALYRENGN----RWTMTERGRGHVERSATQLSIGPSRVF 94
gi|499791021|ref|WP_011471755.      TDPLNHCALNVALYRNTGN----RWTMTERGRRTVARSAPSLVIGPSDLS 94
gi|499823412|ref|WP_011504146.      ADPLNHCALNVGLYHSAGK----RWTMTERARGRVARNLSSLLIGPSDLA 94
gi|499762149|ref|WP_011442883.      ADPLNHCALNVALYHSAGK----RWTMTERSRGRVARNLHSLLIGPSDLA 94
gi|829076993|ref|WP_047310044.      ADPLDHCAINVALYHQAGK----RWSMTERGRGRVARNVNAFLIGPSDLG 94
gi|503269006|ref|WP_013503667.      ADPLDHCAINVALYHQAGK----RWSMTERGRGRVARNVNAFLIGPSDLG 94
gi|499470438|ref|WP_011157078.      ADPLDHCAINVALYHEAGK----RWTMTERGRGRVARNANSFLIGPSDLG 94
gi|501486651|ref|WP_012495141.      ADPLDHCAINVALYHEAGK----RWTMTERGRGRVARNVNSFLIGPSDLG 94
gi|550975051|ref|WP_022723265.      ADPLDHCALNVALYRPGGK----TWTMTERGRGRVARNVHSFLIGPSDLG 94
gi|764538658|ref|WP_044412067.      ADPMNHCALNVALYRPGGN----RWTMTERARGRVARTVNALLVGPSDLS 94
gi|754113006|ref|WP_041748751.      ADPLNHCAVNVAVYRSGGN----RWAMTERPRGAVGRTTDTFTVGPSRLC 105
gi|737680904|ref|WP_035649991.      ADPINHCAINVAIYRSGGN----RWAMTERPREAVSRTIDTFAVGPSNLS 99
gi|503405597|ref|WP_013640258.      AAPEDFVAINLALYGPRGN----RWTMTERRRHSLRAEADSLAIGPSRLA 97
gi|657196689|ref|WP_029312388.      ADPEDYVAINLALYGPRRT----LWAMTERRRRSLIREPGTLGIGPSRLD 97
gi|703385326|ref|WP_033417843.      SEPLDHSAINLALYGKSSH----RWAMTERRRGSVLRAPDVLAIGRSELR 87
gi|653032131|ref|WP_027283892.      GDPHNHCCLNVALYG-RPR----RWAMTDRRRGALARGADFLQIGPSRLD 93
gi|378403832|emb|CCG08948.1|_1      -DPHDHVAINVALYGAPQG-----WTMTERGRRALARDTTRLTIGPSALS 170
gi|754176764|ref|WP_041795395.      -DPHDHVAINVALYGAPQG-----WTMTERGRRALARDTTRLTIGPSALS 86
```

```
gi|25989732|gb|AAN75036.1|_12-      -DPENHVAINVALYNRRGG----RWAMTERGREALVRDDSTLAIGPSALR 178
gi|860504149|ref|WP_048445338.      -DPIDHSAVNVVLYGKPA-----AWAMTERRRHSVARDAASLAIGPSSLD 86
gi|860474721|ref|WP_048434413.      -DPIDHSAVNVVLYGRPK-----AWAMTERRRARVSRTATSLAIGPSALD 86
gi|501291381|ref|WP_012333472.      -DPLDHSAVNVVLYGAPA-----RWAMTERRRRSVARDAASLSIGPSSLA 86
gi|860453591|ref|WP_048427078.      -DPINHSAINVVLYGRPK-----AWAMTERSRRHVNRTATSLAIGPSSLD 73
gi|860560381|ref|WP_048464709.      -DPINHSAVNVVLYGRPK-----AWAMTERSRRHVSRTATSLAIGPSSLD 73
gi|860527577|ref|WP_048454269.      -DPINHSAINVVLYGRPK-----AWAMTERSRRHVSRTATSLAIGPSSLD 86
gi|760871815|dbj|BAQ48812.1|_1      -DPINHSAMNVVLYGRPK-----AWAMTERSRRHVDRTATSLAIGPSSLD 73
gi|373567336|gb|EHP93308.1|_6-      -DPFAHCAVNVVLYGERGN----RFAMTERNARSLSRDATSFSLGPSSLE 93
gi|240009448|gb|ACS40674.1|_4-      -DPFAHCAFNVVLYGERGN----RFAMTERNARSLSRDATSFSLGPSSLE 84
gi|218523206|gb|ACK83791.1|_4-      -DPFAHCAVNVVLYGERGN----RFAMTERNAGSLTRDARSFSLGPSSLE 84
gi|254269168|emb|CAX25134.1|_4      -DPFAHCAVNVVLYGERGN----RFAMTERNARSLTRDATSFSLGPSSLE 84
gi|163663749|gb|ABY31116.1|_2-      -DPFAHCAVNVVLYGERGN----RFAMTERNARSLARDATSFSLGPSSLE 87
gi|494837541|ref|WP_007563641.      -DPFAHCAMNVVLYGDRT-----RFAMTERGAASLSQSRHSIAIGPSAMA 73
gi|914802769|ref|WP_050733395.      -DPFAHCAMNVVLYGDRT-----RFAMTERGAASLSRSRHSVAIGRSAMA 73
gi|657246165|ref|WP_029357447.      -DPFAHCAMNVVLYGDRG-----RFAMTERRAASLSRSADHIAVGPSAMH 73
gi|501276758|ref|WP_012319776.      -DPFAHCAMNVVLYGDRG-----RFAMTERRAASLSRSADHIAVGPSAMH 73
gi|640229117|ref|WP_024829694.      -DPFAHCAMNVVLYGDRG-----RFAMTERRAASLSRSADHIAVGPSAMH 73
gi|518936109|ref|WP_020091984.      -DPFAHCAMNVVLYGDRG-----RFAMTERRATSLSRSADHIAVGPSAMH 73
gi|760075276|ref|WP_043757989.      -DPFAHCAMNVVLYGDRG-----RFAMTERRAASLSRGPDHIAIGPSAMH 73
gi|759662308|ref|WP_043379914.      -DPFAHCAMNVVLYGDRG-----RFAMTERRAASLSRGPDHIAIGPSAMH 73
gi|498368472|ref|WP_010682628.      -DPFAHCAMNVVLYGDRS-----RFAMTERRAASLSRSADHIAVGPSVMH 73
gi|497224411|ref|WP_009538673.      TDPARHCCMNVVLYGASAN----RWAMTERGAASLARDATTIRIGPSGLR 86
gi|589291345|gb|AHK79488.1|_12      --PNHHCAINVALYGDVKR-----WAMTERGRGDLARDSHNLIIGPSAMS 131
gi|644125233|ref|WP_025281955.      --PNHHCAINVALYGDVKR-----WAMTERGRGDLARDSHNLIIGPSAMS 76
gi|496218797|ref|WP_008932834.      --PDHHVSINVALYGNAPR----RWAMTERGRGDLQRDRDNLVIGPSALE 90
gi|495659402|ref|WP_008383981.      VDPHDFCSLNVALYGDCR-----RWAMTERDRGALSRDASSLAIGPSALA 80
gi|687960265|gb|KGE05197.1|_16      -DPEQYCGFNAVLYNPGRK----HWAMTERGAGDLIRDSEHLRARGSAIH 91
gi|496312569|ref|WP_009021747.      QNAENHCAFNTILYGPSGRK---RWSMTERSGARLERHHDSYQLGPSRID 119
gi|495571757|ref|WP_008296336.      TAAENHCAFNTILYGPGGKK---RWSMTERGSARLKRRADSYALGPSRID 95
gi|908380581|ref|WP_049751705.      PNPEDHCAFNVALYGEK-RG---HWSMTERDRHALQRDRDTLIVGPSQAR 77
gi|658524263|ref|WP_029718812.      ADPENHCALNLALYGDAG----KRWTMTERGRRWMRRSRDEFVIGPSRLH 71
gi|332109922|gb|EGJ10550.1|_1-      -------------------------MTERGRRWMRRSRDEFVIGPSRLH 24
gi|760103377|ref|WP_043785587.      ADPENHCALNIALYGDAG----KRWTMTERGRRWMRRSRDEFVIGPSRLH 71
gi|760129436|ref|WP_043810804.      ADPENHCALNIALYGDAG----KRWTMTERGRRWMRRGRDEFVIGPSRLH 71
gi|7416798|dbj|BAA94047.1|_1-4      ADPENHCALNIALYGDAG----KRWTMTERGRRWMRRSRDEFVIGPSRLH 194
gi|75345570|sp|P95619.1|CRTC_R      ADPENHCALNIALYGDAG----KRWTMTERGRRWMRRSRDEFVIGPSRLH 194
gi|913634290|gb|KNZ32951.1|_1-      ADPENHCCLNVALYGPPGQRGAKRWTMTERSRASITREARRFVIGPSRLE 76
gi|809277751|ref|WP_046331236.      ANANDYCAINVALYTPNS----KYWTMTERAQNAINRTEHTFTIGPSHLH 77
gi|518258896|ref|WP_019429104.      ADPDNYCALNVAIYSKGQ----GRWAMTERGQRHCQRSESQFTIGPSQLS 77
gi|518255752|ref|WP_019425960.      ANPDNHCALNVAIYSKGQ----GRWAMTERGKRHCSRTERQFTIGPSQMS 77
gi|918152310|gb|KOF08324.1|_1-      ANPDHHCALNVAIYSKGQ----GRWAMTERGQRHCVRSARQFTIGPSQLS 77
gi|61653243|gb|AAX48196.1|_1-2      MNPDNHCALNVALYSKGK----GRWAMTERGHRHSARSAQAFIIGPSQLA 77
                                              *                :*:*                *

gi|350284778|gb|AEQ27763.1|_1-      WNADGTLTIAIDEIGTP------LPQRVRGRIRVTPLFVNERAFTLDSNG 235
```

```
gi|521992738|ref|WP_020504009.      WSADGTLTIDIDEIGTP------LPQRVRGRIRVTPLFVNERAFTLDSNG 133
gi|557030915|gb|ESQ08988.1|_1-      WHDDETLTVNIKEIGTP------IPQRVRGQVRLFPEFINQRAFALDAAG 123
gi|494353717|ref|WP_007191968.      WDADGGLTVELDEIGTP------IPQRVRGLVKLAPAFINEQVFTLDQAG 123
gi|570733543|gb|AHF04706.1|_24      WNPDQTLTVELDEMGMPFPQR-VVPQRIRGRIHVKPNFINQRVFTLDANG 145
gi|760081160|ref|WP_043763735.      WNPDQTLTVELDEMGMPFPQR-VVPQRIRGRIHVKPNFINQRVFTLDANG 145
gi|528190751|gb|EPY01080.1|_43      WTGS-ALEIRLSERTFP------LPGRIQGTIKVHPRALTGHSVRLDGAG 180
gi|748594904|ref|WP_039853084.      WTGS-ALEIRLSERTFP------LPGRIQGTIKVHPRALTGHSVRLDGAG 133
gi|488815067|ref|WP_002727473.      WTGN-ALEIRLSERTFP------LPGRIQGTIRVHPRALTGHSVRLDGPG 218
gi|505094729|ref|WP_015281831.      WDGE-ALTIDVDEVTAP------IPSRLRGRIRLHPQALVDYPVSLDGKG 120
gi|754165669|ref|WP_041786299.      WDGE-GLVFEIDEATMP------LPGRVRGRVRVRPSAVTSHVETLDAEG 137
gi|739596279|ref|WP_037453842.      WDGT-SLTIRVDETTMP------VPSRVRGTIRVHPPALTPRAFALDDAG 120
gi|764363842|ref|WP_044336870.      WDGD-SLVIDIAEISAPLP------YKVRGTVRVTPEMIGTTAFTLDPEG 136
gi|644239143|ref|WP_025293539.      WDGD-TLVIDIAEISAPLP------YKVRGTVRVSPEMIGTTAFALDPAG 136
gi|503035479|ref|WP_013270455.      WDGG-DLIIRIDEITAPVPPMPTIPKPLRGTVRVRPHAINGTAFPLDAAG 123
gi|617472745|gb|KAI93218.1|_2-      WDGT-RLIVRIDEK-APVTG-----LPVRGVVTLEPEIRSDFATDFGTDG 208
gi|737603811|ref|WP_035574467.      WDGT-ALTIAIDEIAVPIP------QRLRGTVRLFPTALTGHAETLDAAG 119
gi|648530966|ref|WP_026222717.      WRDG-ALTIRVNEIAAPLP------KPIRGEIVVKPLAINTKEFSLESKG 117
gi|288897641|gb|ADC63477.1|_28      WEGD-ALVIDIDEISAP------IPSRLRGRVRVHPSGINGRDFALDAAG 208
gi|760114352|ref|WP_043796307.      WEGD-ALVIDIDEISAP------IPSRLRGRVRVHPSGINGRDFALDAAG 120
gi|494098690|ref|WP_007039487.      WDGD-ALVIDVDEIAAP------IPARIRGRIKVHPEAINTQDFALDADG 120
gi|75388571|sp|Q7X3G5.1|CRTC_T      WDGT-ALTIDVNEITAP------IPSRVRGRIRVIPAAVNAREFTLDPAE 209
gi|390426908|gb|AFL73973.1|_35      WDGT-CLTIRIEEIAVP------IPRRIRGTVKLYPRTVAGQPYFLDGAG 169
gi|752807498|ref|WP_041446922.      WDGT-CLTIRIEEIAVP------IPRRIRGTVKLYPRTVAGQPYFLDGAG 130
gi|760074757|ref|WP_043757486.      WDGS-SLIIDINEITVP------IPSRLRGRVKVYPNAIAARPYQLDHAG 138
gi|918211006|ref|WP_052348303.      WDGS-SLIIDINEITVP------IPSRLRGRVKVYPNAIAARPYQLDHAG 130
gi|499982040|ref|WP_011662758.      WDGN-SLIIHIDEITVP------IPRRIRGTVRVHPTAMLGQSYPLDPAS 139
gi|493840365|ref|WP_006787509.      WQDG-ALLIDINEVTVP------VPSKIRGQVRVYPQAFNPQSFSLDAAQ 123
gi|653037601|ref|WP_027289289.      WDGR-TLTIHLDEIAAP------LPRAVQGRVRVTPHAMTTQEFAIDHQD 146
gi|519009821|ref|WP_020165696.      WDGA-ALTIDLDEIGAP------LPMRVRGSVRLHAPTLAQDAFALDAEG 142
gi|518759539|ref|WP_019916969.      WNGA-ALTIDLDEIGAP------LPMRVRGSVRLHAPTLVQDTFALDADG 138
gi|495340016|ref|WP_008064750.      WDGD-ALTIELDEIGAP------LPLRVRGSVRLHAPTFAQDAFALDADR 138
gi|517223140|ref|WP_018411958.      WEGD-ALVIDLDEIGAP------LPRRVCGQVRLFAPTLRADSFALDSDG 140
gi|889919304|ref|WP_048861895.      WDGD-ALVIDIDERAAP------LPRRVRGRVRVHPGTPTDATFHLDAER 137
gi|496441596|ref|WP_009150441.      WDGD-DLVIDIHETAAP------IPLPLRGQVRVRPQTMTRHVEQLDQHG 212
gi|146409846|gb|ABQ38352.1|_10      WTGR-ELTIEINEVTVP------WPLPLRGKVRVVPTALNEIAFTLDAAG 226
gi|908659074|ref|WP_049795525.      WTGR-ELTIEINEVTVP------WPLPLRGKVRVVPTALNEIAFTLDAAG 133
gi|365289544|emb|CCD93979.1|_1      WNGR-ELSIDINEVSVP------WPLPLRGKVRVVPTALNEVAFTLDAEG 226
gi|41018902|gb|AAR98493.1|_10-      WNGR-ELTIDICEVTVP------WPLPLRGKVRVVPSALNEVAFALDGQQ 226
gi|653027709|ref|WP_027279528.      WDGT-ALNFEIDEVTAP------IPSRIKGRVRVIPRAITQQAFTLNEDG 137
gi|499791021|ref|WP_011471755.      WDGN-ALTININEVTAP------IPGRIRGRVRVIPRAVTQQAFTLNAEG 137
gi|499823412|ref|WP_011504146.      WDGK-ALTININEVCAP------IPGRIRGRVRVIPTTVTKQCFVLNEDG 137
gi|499762149|ref|WP_011442883.      WDRR-ALTININEVRAP------IPGRIKGRVRVIPQFVTKNNFVLNTDG 137
gi|829076993|ref|WP_047310044.      WDGK-TLTINIRETGAP------VPGRIEGRVRVTPAAITKDFFVLNPQG 137
gi|503269006|ref|WP_013503667.      WDGK-TLTINIRETGAP------VPGRIEGRVRVTPAAITKDFFVLNLQG 137
gi|499470438|ref|WP_011157078.      WDGK-TLTINIRETGAP------VPGRIEGRVRVTPVAITKDFFVLNPQG 137
gi|501486651|ref|WP_012495141.      WDGK-TLTINIRETGAP------VPGRIEGRVRVTPVAITKDFFVLNPQG 137
```

```
gi|550975051|ref|WP_022723265.      WDGK-ALTINIRERSAP------IPGRIEGRVRVTPQAITKQVFVLNPDG 137
gi|764538658|ref|WP_044412067.      WDGT-ALTININEVSAP------VPGRIRGRVRVLPSAINSQCFVLNADG 137
gi|754113006|ref|WP_041748751.      WDGK-SLTINVDEISVP------IPGRLRGTIRLIPTAITQQAFLLNGEG 148
gi|737680904|ref|WP_035649991.      WNGS-SLTINVDEISVP------IPGRLRGTIRLVPMAVTPQVFLLNPAG 142
gi|503405597|ref|WP_013640258.      VDAG-GLAAEIAEWSNP------LPRRVRGTVRLVPHALEPTTYKLDAAG 140
gi|657196689|ref|WP_029312388.      WNGN-RLTARITELTTP------IPQPMRGTITLHPKALEPSIYPLDAAA 140
gi|703385326|ref|WP_033417843.      WNGQ-SLVIDLDERTVP------WPSRIRGRLRLHPRALSGHVETLAASG 130
gi|653032131|ref|WP_027283892.      WDGE-ALTIRIQEVTAP------IPSRLSGVVRLRPRALSTRRFELDRAG 136
gi|378403832|emb|CCG08948.1|_1      WTED-GLSVTLDERMAP------VPKPLRGRLTVRPVLQNNRTFFLDAEG 213
gi|754176764|ref|WP_041795395.      WTED-GLSVTLDERMAP------VPKPLRGRLTVRPVLQNNRTFFLDAEG 129
gi|25989732|gb|AAN75036.1|_12-      WDGT-ALRITLNEISFP------KPARVRGEVVIHPEVRTTVPQVLDGAG 221
gi|860504149|ref|WP_048445338.      WDGT-ALTIRVEEVTSP------LPSRLSGTIRVRPEGITPGPFTLDAEG 129
gi|860474721|ref|WP_048434413.      WDGT-VLTIRVDEVTSP------LPSRLSGTIRVRPSGITPGPFTLDAEG 129
gi|501291381|ref|WP_012333472.      WDGS-ALTIRVSEVTAP------LPSRLSGTIRVYPRGITPGPFTLDAEG 129
gi|860453591|ref|WP_048427078.      WDGT-SLTIRVDEVTTP------LPSRLAGTIRVRPGGFTPAPFTLDGEG 116
gi|860560381|ref|WP_048464709.      WDGT-TLTIRVDEVTTP------LPARLTGTIRVRPGGITPAPFTLDAEG 116
gi|860527577|ref|WP_048454269.      WDGT-VLTIRVDEVTTP------LPRRLAGTIRVRPGGFTPAPFTLDAQR 129
gi|760871815|dbj|BAQ48812.1|_1      WDGT-TLTIRIDEVTTP------LPSRLSGTIRVRPGGFTPAPFTLDAER 116
gi|373567336|gb|EHP93308.1|_6-      WDGS-VLTIRLDERSSP------VPRAVRGTVRVRPRALTAQPFTLDTHG 136
gi|240009448|gb|ACS40674.1|_4-      WDGS-VLTIRLDERSSP------VPRAVRGTVRVRPRALTAQPFTLDTHG 127
gi|218523206|gb|ACK83791.1|_4-      WDGS-VLTIRLDERSSP------VPRAVRGTVRVRPRALTAQPFTLDTHG 127
gi|254269168|emb|CAX25134.1|_4      WDGS-VLTIRLDERSSP------VPRAVRGTVRVRPRALTAQPFTLDTHG 127
gi|163663749|gb|ABY31116.1|_2-      WDGS-VLTIRLDERSSP------VPRAVRGTVRVRPRALTAQPFTLDTHG 130
gi|494837541|ref|WP_007563641.      WDGT-TLTIQIDERGAP------LPRRVRGTVRLTPRGFTSGPFQLDPNG 116
gi|914802769|ref|WP_050733395.      WDGT-TLTIRTDERGAP------LPRRVRGTVRLTPRGFTSGPFQLDPNG 116
gi|657246165|ref|WP_029357447.      WDGT-ALTVRIDERGAP------LPRRIRGTVRLEPRGFTGGPFHLDRAG 116
gi|501276758|ref|WP_012319776.      WDGT-ALTVRIDERGAP------LPRRIRGTVRLEPRGFTGGPFHLDRAG 116
gi|640229117|ref|WP_024829694.      WDGT-ALTVRIDERGAP------LPRRIRGTVRLEPRGFTGGPFHLDRAG 116
gi|518936109|ref|WP_020091984.      WDGT-ALTVRIDERGAP------LPRRIRGTVRLEPRGFTGGPFHLDRAG 116
gi|760075276|ref|WP_043757989.      WDGT-ALTVRIDERGAP------LPHRLRGTVRLEPRGFTAGPFHLDRAG 116
gi|759662308|ref|WP_043379914.      WDGT-ALTVRIDERGAP------LPHRLRGTVRLEPRGFTAGPFHLDRAG 116
gi|498368472|ref|WP_010682628.      WDGT-ALTVRIDERGAP------LPHRIRGTVRLEPRGFTGGPFHLDRAG 116
gi|497224411|ref|WP_009538673.      WEGG-VLTVDFDEVTAP------IPGRLKGRLRVHADAVTPRAFTLDPAG 129
gi|589291345|gb|AHK79488.1|_12      WDGK-ALTVTIDETTAP------IPSRLRGQVRLTPHCLTGYSFALDGKG 174
gi|644125233|ref|WP_025281955.      WDGK-ALTVTIDETTAP------IPSRLRGQVRLTPHCLTGYSFALDGKG 119
gi|496218797|ref|WP_008932834.      WGPD-GLTVHLNEITAP------LPSRIRGTIRLRPEALAARSYTLDQQE 133
gi|495659402|ref|WP_008383981.      WDGT-TLTIEIDETATP------LPSRLRGTVRVRPAGLTGKSFALDGAG 123
gi|687960265|gb|KGE05197.1|_16      WQGR-QISVTLDEVTVP------VPGRLRGRIELELPGITATDYALDPAG 134
gi|496312569|ref|WP_009021747.      WDGH-SFLAQVNERCTP------FAQRMQGNIRITPSALTTHSLQLDHRG 162
gi|495571757|ref|WP_008296336.      WDGE-SFLARVNERCTP------FAQRMQGTIRITPSTLTAHSLQLDEDA 138
gi|908380581|ref|WP_049751705.      WVDG-ELLLDIDEITFP------RPAYLRGRIRLTPRVANDQSLVLDAAG 120
gi|658524263|ref|WP_029718812.      WDGE-SLLVEFDEIGVP------IPRRVKGRVRVWPKALCRFVTSLDSGG 114
gi|332109922|gb|EGJ10550.1|_1-      WDGE-SLLVEFDEIGVP------IPRRVKGRVRVWPKALCRFVTSLDSGG 67
gi|760103377|ref|WP_043785587.      WDGE-SLLVEFDEIGVP------IPRRVKGRVRVWPKALCRFVTSLDSGG 114
gi|760129436|ref|WP_043810804.      WDGE-SLLVEFDEIGVP------IPRRVKGRVRVWPKALCRFVTSLDSGG 114
gi|7416798|dbj|BAA94047.1|_1-4      WDGE-SLLVEFDEIGVP------IPRRVKGRVRVWPKALCRFVTSLDSGG 237
```

```
gi|75345570|sp|P95619.1|CRTC_R      WDGE-SLLVEFDEVGVP------IPRRVKGRVRVWPKALCRFVTSLDSGG 237
gi|913634290|gb|KNZ32951.1|_1-      WDGQ-ALTIHVDEVTVP------IPGRVRGTVRVVPQRLFNFSTALDDGA 119
gi|809277751|ref|WP_046331236.      WEND-VLTIEIKERVPL------LGNKVEGTIKVYPDQLFNHVIALDDQE 120
gi|518258896|ref|WP_019429104.      WDGD-CLSIDIDEVCVP------LPRRVRGRIKLWPQQLFGYSTPLDAAG 120
gi|518255752|ref|WP_019425960.      WDGD-CLNIDIDEVCVP------IPRRIRGRIKLWPQQLFAYSTPLDVGG 120
gi|918152310|gb|KOF08324.1|_1-      WDGD-CLHIDIDEVCVP------IPRRVRGHIKLWPQQLFGYATPLDEAG 120
gi|61653243|gb|AAX48196.1|_1-2      WDGD-CLTIDINEVCVP------LPRRIRGRIKLWPQQLCAFSTRLDAFG 120
                                          :     *              : * : :           :

gi|350284778|gb|AEQ27763.1|_1-      RHQWRPIAPSARVEVELSRPDLSWSGHAYFDRNWGTEPLEDAFVYWDWSR 285
gi|521992738|ref|WP_020504009.      RHQWRPIAPSARVEVELSRPDLSWSGHAYFDRNWGTEPLEDAFVYWDWSR 183
gi|557030915|gb|ESQ08988.1|_1-      RHFWRPIAPSARVEVTLQRPALRWSGHAYFDHNWGAEPLEDAFIYWDWSR 173
gi|494353717|ref|WP_007191968.      RHRWRPIAPSAKVEVSFERPALNWTGHAYFDRNWGDEPLEDAFRYWDWSR 173
gi|570733543|gb|AHF04706.1|_24      RHHWRPIAPTARVEVELDRPDLKWSGHAYLDRNWGAEPLEDAFAYWDWCR 195
gi|760081160|ref|WP_043763735.      RHHWRPIAPTARVEVELDRPDLKWSGHAYLDRNWGAEPLEDAFAYWDWCR 195
gi|528190751|gb|EPY01080.1|_43      EHFWSPLAPDSRVEVELSHPRLRWSGPGYFDTNSGIVPLEKSFARWTWSR 230
gi|748594904|ref|WP_039853084.      EHFWSPLAPDSRVEVELSHPRLRWSGPGYFDTNSGIVPLEKSFARWTWSR 183
gi|488815067|ref|WP_002727473.      EHFWSPLAPESRVEVELNHPDLRWSGPGYFDTNSGIVPLEKSFVRWTWSR 268
gi|505094729|ref|WP_015281831.      RHHWRPIAPCSRVEVTMEQPDLRWSGAGYFDTNWGSEPLEDGFACWDWSR 170
gi|754165669|ref|WP_041786299.      RHRWWPVAPASRVEVELDRPSLRWSGAGYFDCNWGDAPLAQDFPDWDWCR 187
gi|739596279|ref|WP_037453842.      RHRWWPISPRCRVEVALDQPGTRWSGTGYLDTNAGDEALEDAFSQWDWCR 170
gi|764363842|ref|WP_044336870.      RHRWHPVAPRAHVEVAMTHPGMRWSGAGYFDSNFGDEPLEAGFNDWHWSR 186
gi|644239143|ref|WP_025293539.      LHRWHPVAPRAHVEVDMSHPGLRWSGAGYFDSNFGDEPIEDGFNDWHWSR 186
gi|503035479|ref|WP_013270455.      KHVWRPIAPRAHVEVTLDDPGLSWTGEGYLDTNGGTEPLEDAFEVWDWSR 173
gi|617472745|gb|KAI93218.1|_2-      RHLWAPIAPRARVEARFDAPAISWTGTGYFDTNRGDEPLEDGFSYWNWSR 258
gi|737603811|ref|WP_035574467.      RHRWRPIAPVCRVEVAMDRPALAWSGHGYWDANEGDEPLEDGFASWTWSR 169
gi|648530966|ref|WP_026222717.      NHIWRPIAPVARVSLDLDAPDLRWKGHGYFDTNAGDEPLERAFAFWTWSR 167
gi|288897641|gb|ADC63477.1|_28      RHRWWPIAPLARVEVDFTNPGLSWKGTGYLDSNRGSEPVERAFQGWDWSR 258
gi|760114352|ref|WP_043796307.      RHRWWPIAPLARVEVDFTNPGLSWKGTGYLDSNRGSEPVERAFQGWDWSR 170
gi|494098690|ref|WP_007039487.      RHRWWPVGPVSRVEVELKHPNLRWSGPGYLDSNRGSEPLEDAFQGWDWSR 170
gi|75388571|sp|Q7X3G5.1|CRTC_T      RHVWWPIAPISRVEVDLEKPALRWSGHGYLDSNRGEEPLEDAFQCWDWSR 259
gi|390426908|gb|AFL73973.1|_35      RHRWAPIAPGARVEVQLDQPALSWAGHGYLDSNWGSEPLEKAFRYWEWSR 219
gi|752807498|ref|WP_041446922.      RHRWAPIAPGARVEVQLDQPALSWAGHGYLDSNWGSEPLEKAFRYWEWSR 180
gi|760074757|ref|WP_043757486.      RHYWAPIAPRAHVEVELDKPSLRWSGSGYLDSNWGVEPLERAFQRWEWSR 188
gi|918211006|ref|WP_052348303.      RHYWAPIAPRAHVEVELDKPSLRWSGSGYLDSNWGVEPLERAFQRWEWSR 180
gi|499982040|ref|WP_011662758.      RHHWEPLAPCAHVEVDLDRPSLRWSGHGYLDSNRGDEPLEAAFKRWDWSR 189
gi|493840365|ref|WP_006787509.      RHHWRPVAPCARVEVALNSPELHWSGPGYLDSNWGDESIEQGFTRWDWSR 173
gi|653037601|ref|WP_027289289.      RHHWWPMAPTAEVSVALNKPALSWHGTGYLDRNRGDEPLEAGFQEWDWAR 196
gi|519009821|ref|WP_020165696.      RHLWLPIAPCARVEVRLSEPALHWHGDAYADSNAGTRALEEDFTRWHWSR 192
gi|518759539|ref|WP_019916969.      RHLWLPIAPCARVEVRLSEPALHWHGDAYADSNAGSRALEEDFTRWHWSR 188
gi|495340016|ref|WP_008064750.      RHLWLPIAPCARVEVRLSEPALQWHGDAYADSNAGSRALEQDFTRWHWSR 188
gi|517223140|ref|WP_018411958.      HHRWQPIAPCARIEVRLDVPAMHWLGDAYCDSNSGDLPLEHSFSHWHWSR 190
gi|889919304|ref|WP_048861895.      RHRWSPIAPCARVEVRFDQPALAWAGDGYFDSNAGSVPLEDSFDHWTWSR 187
gi|496441596|ref|WP_009150441.      RHHWWPIAPDSRVSVQLSHPHLHWSGDGYLDSNWGSEPLEQGFTDWDWSR 262
gi|146409846|gb|ABQ38352.1|_10      RHRWQPIAPCCRVSVDFDHPDLHWRGDGYFDINHGDAPLERDFKSWQWSR 276
gi|908659074|ref|WP_049795525.      RHRWQPIAPCCRVSVDFDHPDLHWRGDGYFDINHGDAPLERDFKSWQWSR 183
```

```
gi|365289544|emb|CCD93979.1|_1      RHRWQPIAPCCRVSVDFDHPDLHWRGDGYFDLNHGDAPLERDFQSWQWSR 276
gi|41018902|gb|AAR98493.1|_10-      RHRWQPIAPCCRVSVDFDHPDLHWRGDGYFDINHGDAPLEHDFQSWQWSR 276
gi|653027709|ref|WP_027279528.      NHRWWPIAPCSRVEVELEKPKLRWSGEGYFDMNAGDVPIEHGFTVWEWSR 187
gi|499791021|ref|WP_011471755.      NHRWWPIAPSARVEVEMAAPDLAWQGEGYFDMNAGDVPLEQGFTVWEWSR 187
gi|499823412|ref|WP_011504146.      NHRWWPIAPRSRIEVTLDQPKLNWQGEAYIDTNAGDAPIETGFSHWEWAR 187
gi|499762149|ref|WP_011442883.      NHRWWPIAPRSRIEVTLEQPKLSWQGEAYLDCNAGDAPIETGFTHWEWAR 187
gi|829076993|ref|WP_047310044.      NHRWWPIAPRARVEVDLRRPNLSWQGEGYLDSNAGDEPIETGFRHWEWAR 187
gi|503269006|ref|WP_013503667.      NHRWWPIAPRARVEVDLRRPNLSWQGEGYLDSNAGDEPIETGFRHWEWAR 187
gi|499470438|ref|WP_011157078.      NHRWWPIAPRARVEVDLRRPNLSWRGEGYLDTNAGDEPIETGFRHWEWAR 187
gi|501486651|ref|WP_012495141.      NHRWWPIAPRARVEVDLRRPNLSWRGEGYLDTNAGDEPIETGFRHWEWAR 187
gi|550975051|ref|WP_022723265.      NHRWWPIAPRSRIEVDLRRPDLSWRGEAYLDTNAGDAPIETGFKHWEWAR 187
gi|764538658|ref|WP_044412067.      NHRWWPIAPCARVEVEMAQPSLRWQGEGYLDTNAGDAPIETGFRDWEWSR 187
gi|754113006|ref|WP_041748751.      NHQWWPIAPCARVQVRLDHPRLSWQGDGYFDMNHGDTPLEQGFSDWQWSR 198
gi|737680904|ref|WP_035649991.      RHQWWPIAPFARVQVRLDHPHLSWQGDGYLDMNHGDEPLEQGFSDWQWAR 192
gi|503405597|ref|WP_013640258.      RHRWQPLAPCARIRVAFTAPELAWEGNAYLDRNDGDEPIAHAFRRWHWSR 190
gi|657196689|ref|WP_029312388.      AHRWQPIAPCARISVTFEKPALSWEGDAYFDRNEGDAPMHTAFSTWNWSR 190
gi|703385326|ref|WP_033417843.      HHVWTPIAPSARIEVELDSPKLSWSGEAYLDGNAGDTPIEDAFRSWHWSR 180
gi|653032131|ref|WP_027283892.      RHLWHPIAARARVEVALENPALRWSGPAYFDWNAGAAPLEQDFREWDWCR 186
gi|378403832|emb|CCG08948.1|_1      RHRWRPVWPRARIEVDLDAPAARWSGSAYFDTNDGDEPMQDAFQRWDWSR 263
gi|754176764|ref|WP_041795395.      RHRWRPVWPRARIEVDLDAPAARWSGSAYFDTNDGDEPMQDAFQRWDWSR 179
gi|25989732|gb|AAN75036.1|_12-      RHVWWPFAPRSRVELRMDNPDIRWNGHGYFDFNCGSEPLQDAFSRWDWSR 271
gi|860504149|ref|WP_048445338.      RHRWWPLSPAARIEVAFDRPSLHWQGHAYLDTNEGDVPLEHSFRSWTWSR 179
gi|860474721|ref|WP_048434413.      RHRWWPLAPSSRIEVALDRPSLRWNGHAYFDTNDGDVALEDSFRSWTWSR 179
gi|501291381|ref|WP_012333472.      RHRWWPIAPASRIEVDLDRPALRWRGHAYFDTNDGDTPLEASFRSWTWSR 179
gi|860453591|ref|WP_048427078.      RHRWWPLAPSSRVEVAFDKPSLRWSGHAYFDTNEGDVPLEDSFRSWTWSR 166
gi|860560381|ref|WP_048464709.      HHRWWPLAPTSRVEVAFDRPSLRWSGHAYFDTNEGDVPLEASFKSWTWSR 166
gi|860527577|ref|WP_048454269.      HHRWWPLAPSSRVEVAFDRPSLNWNGHAYFDTNDGDVPLEQSFKSWTWSR 179
gi|760871815|dbj|BAQ48812.1|_1      RHRWWPLAPSSRVEVAFGRPGLRWSGHAYFDTNDGDVPLEDSFRSWTWSR 166
gi|373567336|gb|EHP93308.1|_6-      LHRWWPMAPDCEVEAAFTAPALSWRGSGYHDTNDGDGALEDAFTDWTWCR 186
gi|240009448|gb|ACS40674.1|_4-      LHRWWPMAPDCEVEAAFTAPALSWRGSGYHDTNDGDGALEDAFTDWTWCR 177
gi|218523206|gb|ACK83791.1|_4-      LHRWWPMAPDCEVETEFTAPALSWRGSGYHDTNDGDGALEDAFTDWTWCR 177
gi|254269168|emb|CAX25134.1|_4      LHRWWPMAPDCEVEAAFTAPALSWRGSGYHDSNDGDGALEEAFTEWTWCR 177
gi|163663749|gb|ABY31116.1|_2-      LHRWWPMAPDCEVEAEFTAPTLSWRGSGYHDTNDGDGALEDAFTDWTWCR 180
gi|494837541|ref|WP_007563641.      RHRWWPMAPSSRVEVELDQPNLRWTGSGYFDTNDGDEPLASAFIRWTWCR 166
gi|914802769|ref|WP_050733395.      RHRWWPMAPSSRVEVELDQPNLRWTGSGYFDTNDGDEPLGSAFTRWTWCR 166
gi|657246165|ref|WP_029357447.      RHRWWPMSPSSRVEVDFDQPDLRWSGSGYFDTNDGDEPLEAAFARWTWCR 166
gi|501276758|ref|WP_012319776.      RHRWWPMSPSSRVEVDFDQPDLRWSGSGYFDTNDGDEPLEVAFARWTWCR 166
gi|640229117|ref|WP_024829694.      RHRWWPMSPSSRVEVDFDQPDLRWSGSGYFDTNDGDEPLEAAFARWTWCR 166
gi|518936109|ref|WP_020091984.      RHRWWPMSPSSRVEVDFDAPDLRWSGSGYFDTNDGDEPLEAAFSRWTWCR 166
gi|760075276|ref|WP_043757989.      RHRWWPMAPSSRVEVDFDQPDLRWSGGGYFDTNDGDEPLEAAFSRWTWCR 166
gi|759662308|ref|WP_043379914.      RHRWWPMAPSSRVEVDFDQPDLRWSGGGYFDTNDGDEPLEAAFSRWTWCR 166
gi|498368472|ref|WP_010682628.      RHRWWPMAPSSRVEVAFDQPGLSWSGSGYFDTNDGDEPLGAAFARWTWCR 166
gi|497224411|ref|WP_009538673.      AHRWWPIAPSSRVEVVLEKPALSWKGRAYFDTNDGSEPLEKGFKDWDWCR 179
gi|589291345|gb|AHK79488.1|_12      LHQWRPIAPSARVEVDLEQPGLQWEGRGYLDSNRGEEPVEKGFRYWDWSR 224
gi|644125233|ref|WP_025281955.      LHQWRPIAPSARVEVDLEQPGLQWEGRGYLDSNRGEEPVEKGFRYWDWSR 169
gi|496218797|ref|WP_008932834.      RHHWRPIAPCARVEVDLQQPGLRWLGHGYMDSNIGTEPLEQGFRYWDWSR 183
```

```
gi|495659402|ref|WP_008383981.      LHRWRPIAPCSDVEVDLARPGLSWRGHGYLDSNIGDEPVESAFVRWDWSR 173
gi|687960265|gb|KGE05197.1|_16      RHRWWPVAPSCRARVTMEKPGLSWEGHGYFDSNRGAEPLEAAFRDWDWSR 184
gi|496312569|ref|WP_009021747.      RHHWNPIAPIARVEVDIPSLDTQWSGDGYLDSNHGSEPLARGFSGWDWTR 212
gi|495571757|ref|WP_008296336.      RHHWNPIAPIARVDVDLPSLGTAWSGDGYLDSNRGTEPLADGFSGWDWTR 188
gi|908380581|ref|WP_049751705.      RHRWCALAPCARVEVEMEKPRVRWQGEGYLDSNAGDEPLEDGFSGWHWSR 170
gi|658524263|ref|WP_029718812.      RHRWGPIAPCSRIEVELDSPRVRWSGHAYLDSNEGDEPIDRPFHEWDWSR 164
gi|332109922|gb|EGJ10550.1|_1-      RHRWGPIAPCSRIEVELDSPRVRWSGHAYLDSNEGDEPIDRPFHEWDWSR 117
gi|760103377|ref|WP_043785587.      RHRWGPIAPCSRIEVELDSPNARWSGHAYVDSNEGDEPIDRPFREWDWSR 164
gi|760129436|ref|WP_043810804.      RHRWGPIAPCSRIEVELDSPNARWSGHAYVDSNEGDEPIDRPFREWDWSR 164
gi|7416798|dbj|BAA94047.1|_1-4      RHRWGPIAPCSRIEVELDSPNARWSGHAYVDSNEGDEPIDRPFREWDWSR 287
gi|75345570|sp|P95619.1|CRTC_R      RHRWGPIAPCSRIEVELDSPRVRWSGHAYLDSNEGDEPIDRPFREWDWSR 287
gi|913634290|gb|KNZ32951.1|_1-      RHHWGPIAPSARVEVAFDSPGTRWSGHAYLDSNEGTEPIERPFTEWDWSR 169
gi|809277751|ref|WP_046331236.      KHRWGPISPSARVEVSFTKPNLHWKGSAYFDSNEGDEAVSQSFSEWDWSR 170
gi|518258896|ref|WP_019429104.      RHRWGPLAPASRIEVDLNAPDLKWQGHAYLDSNEGDEPVDRGFHNWDWSR 170
gi|518255752|ref|WP_019425960.      RHRWGPLAPSSRIEVNLSAPDLKWQGHAYLDSNEGDEPVEQGFHTWDWSR 170
gi|918152310|gb|KOF08324.1|_1-      RHRWGPLAPASRIEVALDAPDLKWQGHAYLDSNEGDEPVERGFHTWDWSR 170
gi|61653243|gb|AAX48196.1|_1-2      RHRWGPLAPASRIEVELSSPDLKWQGHAYLDSNEGDEPIDQGFHTWDWSR 170
                                     * * .. . .     :      * * .* * * *  .:   *  * * *

gi|350284778|gb|AEQ27763.1|_1-      ASMG----EESAILYHVDRR-----SDGP--LSLALRFAADGTLSEFEPP 324
gi|521992738|ref|WP_020504009.      ASMG----EESAILYHVDRR-----GDGP--LSLALRFAADGTLSEFESP 222
gi|557030915|gb|ESQ08988.1|_1-      AGMDG---AESAILYNIDRR-----DGTP--LSLALHFANDGSLSEFEPP 213
gi|494353717|ref|WP_007191968.      ASLGG---DESLILYHADRR-----EGGR--LSLGLHFGADGGLSHFDAP 213
gi|570733543|gb|AHF04706.1|_24      ATLGE---DESAVLYNMDRR-----DGTP--FSLALNFAADGTISEFDPP 235
gi|760081160|ref|WP_043763735.      ATLGE---DESAVLYNMDRR-----DGTP--FSLALNFAADGTISEFDPP 235
gi|528190751|gb|EPY01080.1|_43      APLQ----NGTAVLYDVTRR-----DGSD--LAVALRIGRDGKVEESDSP 269
gi|748594904|ref|WP_039853084.      APLQ----NGTAVLYDVTRR-----DGSD--LAVALRIGRDGKVEESDSP 222
gi|488815067|ref|WP_002727473.      APLK----DGTAILYDVTRR-----DGSD--LAVALRIGHDGKVEESDSP 307
gi|505094729|ref|WP_015281831.      TGLD----DGAAILYDVTRR-----DAER--MSLALRIAGNGEVEAFEPP 209
gi|754165669|ref|WP_041786299.      APVGPQGRDGAAIVYDVRRR-----DGSG--KVIAIRIDRAGQVESFAVP 230
gi|739596279|ref|WP_037453842.      ADFG----GDSAVLYNVDRR-----VGSH--LALALMIGQDGTVEPFEPL 209
gi|764363842|ref|WP_044336870.      AHLK----NDVAVLYEGRRR-----DGTP--FDLALKFDRQGRWHDVVQP 225
gi|644239143|ref|WP_025293539.      AHLK----NDVAVLYEGRRR-----DGTP--FDLALRFDRQGRWHDVVQP 225
gi|503035479|ref|WP_013270455.      AHLK----SDSAVLYDLKRK-----DGSA--LSLALKFDRHGVPETVEPP 212
gi|617472745|gb|KAI93218.1|_2-      ACLS----DSAVMLYEGTRQ-----NGSK--FNMGIQIAADGSVSPVELR 297
gi|737603811|ref|WP_035574467.      APVG----AGAAVLYDAIRR-----DGSA--VSLALRIRADGRVTPAEAP 208
gi|648530966|ref|WP_026222717.      ANIG----DDAAILYDAERR-----REAP--LSLALRFSASGEMETLDPP 206
gi|288897641|gb|ADC63477.1|_28      AELKHG----AAILYDTRAR-----DDQG--AHLALRFDANAEVDEFALP 297
gi|760114352|ref|WP_043796307.      AELKHG----AAILYDTRAR-----DDQG--AHLALRFDANAEVDEFALP 209
gi|494098690|ref|WP_007039487.      AELKQG----AAILYDVRTR-----EGRGPGAELALRCDHRGQLDRFDPP 211
gi|75388571|sp|Q7X3G5.1|CRTC_T      ANTPSG----TTMLYDVTAR-----HGTG--ASLALRFNASGEVEEFPPP 298
gi|390426908|gb|AFL73973.1|_35      AELKDGG---TAVLYDAARR-----DGHR--TELGLHFDPAGDVERIDVP 259
gi|752807498|ref|WP_041446922.      AELKDGG---TAVLYDAARR-----DGHR--TELGLHFDPAGDVERIDVP 220
gi|760074757|ref|WP_043757486.      AMLSDGG---AAILYDVTQR-----EGHR--PPLGLRFAANGDVEHFEAP 228
gi|918211006|ref|WP_052348303.      AMLSDGG---AAILYDVTQR-----EGHR--PPLGLRFAANGDVEHFEAP 220
gi|499982040|ref|WP_011662758.      TALKHG----AAVLYDVTGR-----EGPG--PVLGLRFDPNGDVEPFEAP 228
```

```
gi|493840365|ref|WP_006787509.      AELKDG----TAILYDITRR-----NGEA--FALALKYHPDSGLTQLPPP 212
gi|653037601|ref|WP_027289289.      APLPSGG---TAITYDTFRR-----DGSD--HALSLLVRKDGGIERFDNA 236
gi|519009821|ref|WP_020165696.      SNLRGGD---TAVLYDVQRR-----DGTP--HTLALHFDADGGCRSFAAP 232
gi|518759539|ref|WP_019916969.      SNLRGGD---TAVLYDVERR-----DGTP--HSLALHFDADGGCRSFAAP 228
gi|495340016|ref|WP_008064750.      SSLRGGD---TAVLYDVQRR-----DGTP--HSLALHFDADGGCRHFAAP 228
gi|517223140|ref|WP_018411958.      CTLRGGD---TAVLYDVSGR-----DGAR--TALALHFDADGAQRRFDAP 230
gi|889919304|ref|WP_048861895.      APLRDG----AAILYDVRRR-----DGSV--QNLSLRVGRDGRVADYAAP 226
gi|496441596|ref|WP_009150441.      AKRRQGG---AVVLYEANRR-----DGSA--LSLALQVNRDGSVEPFEPP 302
gi|146409846|gb|ABQ38352.1|_10      AATRTG----TVISYDTTAR-----TGAG--KSIALHIDPDARLDRLAPL 315
gi|908659074|ref|WP_049795525.      AATRTG----TVISYDTTAR-----TGAG--KSIALHIDPDARLDRLAPL 222
gi|365289544|emb|CCD93979.1|_1      AATRTG----TVISYDTVAR-----AGTD--KSIALHVDPDARLRRIAPL 315
gi|41018902|gb|AAR98493.1|_10-      AATRSG----AVISYDAIAR-----DHAI--SPLALHVDHTGRLEPIAPL 315
gi|653027709|ref|WP_027279528.      AALRNG----TAILYEAERR-----DGSR--LDLALQFDKSGAMQMFDFP 226
gi|499791021|ref|WP_011471755.      AALRDG----TAILYEAARR-----DGTR--FDLAIKFDGNGEMEHFEAP 226
gi|499823412|ref|WP_011504146.      GALRDK----TAILYEAERR-----DGSR--LDLAVTFDAQGNMERFAQP 226
gi|499762149|ref|WP_011442883.      GALRDK----TAILYEAERR-----DGSR--VDLAITFDAQGNMERFTPP 226
gi|829076993|ref|WP_047310044.      GAMRDK----TVILYEAERR-----DGSR--VDLAVTFDADGNKQEFTPP 226
gi|503269006|ref|WP_013503667.      GAMRDK----TVILYEAERR-----DGSR--VDLAVTFDADGNKQEFTPP 226
gi|499470438|ref|WP_011157078.      GAMRDK----TVILYEAERR-----DGSR--VDLAVTFDAEGNKQEFTPP 226
gi|501486651|ref|WP_012495141.      GAMRDK----TVILYEAERR-----DGSR--VDLAVTFDAEGNKQEFTPP 226
gi|550975051|ref|WP_022723265.      GALRDK----TAILYEAERR-----DGSR--IDLAMTFDAQGNMQPFTPP 226
gi|764538658|ref|WP_044412067.      GALRDG----TAILYEAERR-----DGSR--FDLAVTFDAKGNMQPFERP 226
gi|754113006|ref|WP_041748751.      GAMQDG----TAILYEAQRR-----DGSR--IDLAMTFDPNGRMQTFEPP 237
gi|737680904|ref|WP_035649991.      GAMQDG----TAILYEAERR-----DGSR--VDLAMTFDRNGRMQAFEPP 231
gi|503405597|ref|WP_013640258.      AATRQG----AAILYHAERR-----DGGQ--TSLALGFAAGGGATAFTPP 229
gi|657196689|ref|WP_029312388.      APSRQG----ARVFYHANRR-----DGGT--TALSLGFTAAGGAAPLHAP 229
gi|703385326|ref|WP_033417843.      NPLRDG----SAVLYDVARR-----DGSA--LNLALRFDGHGEVSRFEPP 219
gi|653032131|ref|WP_027283892.      APMADR----TAILYHAERR-----DGSS--QSLALRVDEDGAVGAFPPP 225
gi|378403832|emb|CCG08948.1|_1      GDLADGG---CALLYDLTPRPH---RGPA--RLLSLRLTPEGQWEEVAPP 305
gi|754176764|ref|WP_041795395.      GDLADGG---CALLYDLTPRPH---RGPA--RLLSLRLTPEGQWEEVAPP 221
gi|25989732|gb|AAN75036.1|_12-      APLADGG---AALLYEVTPR-----QGAD--RLLSLRVDRDGALSAFSPP 311
gi|860504149|ref|WP_048445338.      STLRHG----AAILYDVLRR-----DGSR--QDLSLRFDPDGTPRPIEPP 218
gi|860474721|ref|WP_048434413.      STLRHG----AAILYDVLRR-----DGSR--QDLSLRFDPDGTPRPIAPP 218
gi|501291381|ref|WP_012333472.      ATLRHG----AAILYDVLRR-----DGTR--QDLSLRFDPDGTPRPIEPP 218
gi|860453591|ref|WP_048427078.      ATLTHG----AAILYDVERR-----DGSR--QDLSLRFDPDGTPRPIAPP 205
gi|860560381|ref|WP_048464709.      ATLTHG----SAILYDVLRR-----DGSR--QDLSLRFDPDGTPRPIAPP 205
gi|860527577|ref|WP_048454269.      ATLRHG----AAILYDVERR-----DGSR--QDLSLRFDPDGTPRPIEPP 218
gi|760871815|dbj|BAQ48812.1|_1      ATLRHG----AAILYDVARR-----DGSR--QELSLRFDPDGTPRPIEPP 205
gi|373567336|gb|EHP93308.1|_6-      APLKRG----SAILYDVRRR-----DGSG--QNLTLRFDADGTRREMRPP 225
gi|240009448|gb|ACS40674.1|_4-      APLKRG----SAILYDVRRR-----DGSG--QNLTLRFDADGTRREMRPP 216
gi|218523206|gb|ACK83791.1|_4-      APLKRG----SAILYDVRRS-----DGSG--QNLTLRFDADGTRREMRPP 216
gi|254269168|emb|CAX25134.1|_4      APLKRG----SAILYDVRRS-----DGSG--QNLTLRFDADGTRREMRPP 216
gi|163663749|gb|ABY31116.1|_2-      APLKRG----SAILYDVRRR-----DGSG--QNLTLRFDADGTRREMRPP 219
gi|494837541|ref|WP_007563641.      ADLPDG----AAILYDVRHR-----DGGG--QNLSLRFCHDGNRREIRPP 205
gi|914802769|ref|WP_050733395.      ADLPDG----AAILYDVRHR-----NGGG--QNLSLRFSHDGSRREIRPP 205
gi|657246165|ref|WP_029357447.      ADLPDG----AAILYDVHHR-----DGGG--QNLSLRFSADGSRREIRPP 205
```

```
gi|501276758|ref|WP_012319776.      ADLPDG----AAILYDVHHR-----DGGG--QNLSLRFSADGSRREIRPP 205
gi|640229117|ref|WP_024829694.      ADLPDG----AAILYDVHHR-----DGGG--QNLSLRFSADGSRREIRPP 205
gi|518936109|ref|WP_020091984.      ADLPDG----AAILYDVHHR-----DGGG--QNLSLRFSADGSRREIRPP 205
gi|760075276|ref|WP_043757989.      ADLPDG----AAILYDVHHR-----DGGG--QNLSLRFSADGSRREIRPP 205
gi|759662308|ref|WP_043379914.      ADLPDG----AAILYDVHHR-----DGGG--QNLSLRFSADGSRREIRPP 205
gi|498368472|ref|WP_010682628.      ADLQDG----AAILYDVQHR-----EGGG--QNLSLRFSSDGSRREIRPP 205
gi|497224411|ref|WP_009538673.      APLKDGG---AAILYETRPL-----DGPS--RCLSLRIGRDGSSREIEPP 219
gi|589291345|gb|AHK79488.1|_12      AVMEDQE---TVVLYEADRR-----DGGH--SLLSLRFDADGKAREVEAP 264
gi|644125233|ref|WP_025281955.      AVMEDQE---TVVLYEADRR-----DGGH--SLLSLRFDADGKAREVEAP 209
gi|496218797|ref|WP_008932834.      AVMPNGD---TVVLYEATQR-----DDQE--RMLTLRFDPRGGVRDVEPP 223
gi|495659402|ref|WP_008383981.      AALPDGS---AAVLYHAIRRGADGGEGAE--TGLALKFDRSGAVTEFDAP 218
gi|687960265|gb|KGE05197.1|_16      APLADGG---SLVQYHARLR-----DGSE--RLLALRFGATGAVSELDPL 224
gi|496312569|ref|WP_009021747.      ARLNNGD---CAVRYEARYP-----GLEP--HRLALRFAQDGSISAEPSQ 252
gi|495571757|ref|WP_008296336.      ARLSNGD---CAVRYEARHG-----ARKP--HRLALRFKKDGSIASEETR 228
gi|908380581|ref|WP_049751705.      ASDTDG----TTVLYDVTPR-----QGAP--YRKAIHFDHQGTVQETPAP 209
gi|658524263|ref|WP_029718812.      ATMADGS---TAVIYDVRQK-----RDGD--RVIAERFLLDGSTESFEAP 204
gi|332109922|gb|EGJ10550.1|_1-      ATMADGS---TAVIYDVRQK-----RDGD--RVIAERFLLDGSTESFEAP 157
gi|760103377|ref|WP_043785587.      ATMADGS---TAVIYDVRQK-----RDGD--RVIAERFLLDGSTESFEAP 204
gi|760129436|ref|WP_043810804.      ATMADGS---TAVIYDVRQK-----RDGD--RVIAERFLLDGSTESFEAP 204
gi|7416798|dbj|BAA94047.1|_1-4      ATMADGS---TAVIYDVRQK-----RDGD--RVIAERFLLDGSTESFEAP 327
gi|75345570|sp|P95619.1|CRTC_R      ATMADSS---TAVIYDVRQK-----RDGD--RVIAERFLLDGSTESFEAP 327
gi|913634290|gb|KNZ32951.1|_1-      CSLRDGS---TAVIYDVRQK-----QGAD--RVLALRFTQDGEVEPFEPP 209
gi|809277751|ref|WP_046331236.      AHLKDGS---TAVIYDVRQS-----NGAE--RIIASKFNVDGTVEPFAAP 210
gi|518258896|ref|WP_019429104.      ARTRDGS---TVVFYDMQAP-----ATED--RVLSLRFTPRGTVEPIATP 210
gi|518255752|ref|WP_019425960.      ARMRDGS---TVVFYDMQAP-----GTED--RVLSLRFSPKGTVEPMATP 210
gi|918152310|gb|KOF08324.1|_1-      ARMRDGS---TVVFYDMQAP-----GAED--RVLSLLFTPQGAVEPIAAP 210
gi|61653243|gb|AAX48196.1|_1-2      ARMRDGS---TLVFYDMQAP-----HTQD--RVLSLRFTPKGKVETIDTP 210
                                                : *.                      .

gi|350284778|gb|AEQ27763.1|_1-      PNHRLPRTPIWQVARSSQADADH----PPKIVKTLEDTPFYSRSVIASHL 370
gi|521992738|ref|WP_020504009.      PNHRLPRTPIWQVARSSQADADH----PPRIVKTLEDTPFYSRSVIASHL 268
gi|557030915|gb|ESQ08988.1|_1-      PNHRLPRTPIWRVARSSQADAGH----PPRVVKTLEDTPFYSRSVIDSHL 259
gi|494353717|ref|WP_007191968.      PDHRLARTPIWRMPRSSQADAKH----PPRVVKTLEDTPFYSRSVIASHL 259
gi|570733543|gb|AHF04706.1|_24      PNHKLPRGTVWRSPRSMQADE------PPRIVETLEDTPFYTRSVVSAKL 279
gi|760081160|ref|WP_043763735.      PNHKLPRGTVWRSPRSMQADE------PPRIVETLEDTPFYTRSVVSAKL 279
gi|528190751|gb|EPY01080.1|_43      PMSALPRG-AWGVERVSRADSGH----TARLAEVLEDTPFYTRSVIDTRL 314
gi|748594904|ref|WP_039853084.      PMSALPRG-AWGVERVSRADSGH----TARLAEVLEDTPFYTRSVIDTRL 267
gi|488815067|ref|WP_002727473.      PVADLPRG-VWGVDRATRADSGH----AAQLAEILEDTPFYTRSMVETRL 352
gi|505094729|ref|WP_015281831.      PPAPLATTSVWRIARATQADTGQ----PARVVETLEDTPFYARSVIATHL 255
gi|754165669|ref|WP_041786299.      PRVALPGSFWWRINRGVRCESGG----TAAVRETLVDAPFYARSVVATRM 276
gi|739596279|ref|WP_037453842.      PRVTLPASRWWRIPRPTRADPGQ----PVTVTETLVDAPFYARSVLATHI 255
gi|764363842|ref|WP_044336870.      TAARLPRT-GWLVDRATRVDAGH----RPRVTKTWIDAPFYARSALSTHL 270
gi|644239143|ref|WP_025293539.      APARLPRT-GWLVNRQTRADSGA----APRILKTWIDAPFYARSSIATRL 270
gi|503035479|ref|WP_013270455.      PHAALPAT-LWRMPRHTRADADR----PVSVERTWEDTPFYSRSTIRTHL 257
gi|617472745|gb|KAI93218.1|_2-      ERCDLPLTRYWKMPRETRVDAGH----TANVEQTLEDTPFYSRSMIATHL 343
gi|737603811|ref|WP_035574467.      PEAGLPGT-LWRIPRRTRADADA----TPGVLATLEDAPFYARSTLATRL 253
```

```
gi|648530966|ref|WP_026222717.      PLAPLPLT-KWRVQRRTRADDG-----VAQALRSFEDAPFYSRSLVAAKF 250
gi|288897641|gb|ADC63477.1|_28      PRAPLPTTSVWRIERGIQCEAGDE----PRVVTTLEDTPFYARSVVETRL 343
gi|760114352|ref|WP_043796307.      PRAPLPTTSVWRIERGIQCEAGDE----PRVVTTLEDTPFYARSVVETRL 255
gi|494098690|ref|WP_007039487.      PRVPLQTTRVWRIGRGTQSEPGHE----TRVVDTLEDTPFYARSVIESQL 257
gi|75388571|sp|Q7X3G5.1|CRTC_T      PRVRLPTTGIWRIKRGTQCEAGHQ----ARVVETLEDTPFYARSLVETRL 344
gi|390426908|gb|AFL73973.1|_35      PHATLPTT-AWLMQRGTRVDPQQT----ARVTETLENTPFYTRSVLETHL 304
gi|752807498|ref|WP_041446922.      PHATLPTT-AWLMQRGTRVDPQQT----ARVTETLENTPFYTRSVLETHL 265
gi|760074757|ref|WP_043757486.      PHVRLPNT-SWLMRRGTRVDANKF----ARVAETLEDTPFYTRSVLESHL 273
gi|918211006|ref|WP_052348303.      PHVRLPNT-SWLMRRGTRVDANKF----ARVAETLEDTPFYTRSVLESHL 265
gi|499982040|ref|WP_011662758.      PRIKLPNT-LWRVERETRVDHQSS----ARVVATLEDTPFYARSVLETNL 273
gi|493840365|ref|WP_006787509.      PAHPLPSGPIWRAGRNMQADEGHQ----PRIAKTFEDTPFYARSMVHSQL 258
gi|653037601|ref|WP_027289289.      VLNDLPSTSIWRIRRKGRADAGHR----ACVSKTLEDTPFYARSLVETHL 282
gi|519009821|ref|WP_020165696.      AAHELPG-TRWGIARATRSEGGTT----PQAR-TLEDTPFYARSLLDTCL 276
gi|518759539|ref|WP_019916969.      AAHELPG-TRWGIARATRSEGGTT----PQAR-TLEDTPFYARSLLDTCL 272
gi|495340016|ref|WP_008064750.      AERTLPG-TRWGIARATRSEADTS----PQVR-TLEDTPFYARSLLDTRL 272
gi|517223140|ref|WP_018411958.      PECTLPG-TRWGIARSTRCERGAP----PQVR-TLEDTPFYARSLLHTQL 274
gi|889919304|ref|WP_048861895.      AACPLPG-TRWGIARATRAETPGG----TRLRTTLTDAPFYARSLLDTRI 271
gi|496441596|ref|WP_009150441.      ARVPLPTTTVWRIARATRSEHPVTGETRAQVLETLEDTPFYARSTIATRL 352
gi|146409846|gb|ABQ38352.1|_10      ADAPLRRT-LWRIDRSARADSETP----VRVVSTLEDAPFYARSHLAARI 360
gi|908659074|ref|WP_049795525.      ADAPLRRT-LWRIDRSARADSETP----VRVVSTLEDAPFYARSHLAARI 267
gi|365289544|emb|CCD93979.1|_1      AESPLRKT-LWRVDRSARADAETS----VRVVSTLEDAPFYARSHLAARI 360
gi|41018902|gb|AAR98493.1|_10-      VDCRLPKT-LWRVERSARADAGAP----VRVVSTLEDAPFYARSHLAARV 360
gi|653027709|ref|WP_027279528.      TLHPLPRT-PWRVSRSARSQTE------AAVVRTLEDSPFYARSMIATTL 269
gi|499791021|ref|WP_011471755.      ALERLQRT-PWRVDRSARSEAG------ARVVKTLEDSPFYARSMIATTL 269
gi|499823412|ref|WP_011504146.      PLMTLPRS-GWKVSRSARSERD------AKVLRTLEDTPFYARSMVATTL 269
gi|499762149|ref|WP_011442883.      PLLPLPRS-GWQVSRSARSETD------ARVIRTLEDSPFYARSMVATTL 269
gi|829076993|ref|WP_047310044.      QLLPLPRS-GWKVDRSARSETG------AKLVRTLEDTPFYARSMVETTL 269
gi|503269006|ref|WP_013503667.      QLLPLPRS-GWKVDRSARSETG------AKLVRTLEDTPFYARSMVETTL 269
gi|499470438|ref|WP_011157078.      QLLPLPRS-GWKVDRSARSEAS------VKLVRTLEDTPFYARSMVETTL 269
gi|501486651|ref|WP_012495141.      QLLPLPRS-GWKVDRSARSEAS------VKLVRTLEDTPFYARSMVETTL 269
gi|550975051|ref|WP_022723265.      PLLPLARS-GWKVARSARSETD------ARLLRTLEDTPFYARSMIDTTL 269
gi|764538658|ref|WP_044412067.      QLVPLARS-GWRVNRNARSDNG------ARVVKTLEDAPFYTRSVVATTL 269
gi|754113006|ref|WP_041748751.      PTIDLRRT-GWRVARSARSDSA------AKIVKTLEDAPFYARSVVSASL 280
gi|737680904|ref|WP_035649991.      PTVKLRRT-GWRVARSVRSDGA------AKIVKTLEDAPFYARSVLSAKL 274
gi|503405597|ref|WP_013640258.      PAAALPAT-RWRLARETRSDGGAS------LIRNLEDTPFYARARIGASL 272
gi|657196689|ref|WP_029312388.      PEAPLPIT-WWRINRATRCDADAE----PRLLRAFEDTPFYARARIATTI 274
gi|703385326|ref|WP_033417843.      PAVVLPKT-GWRIDRASRGEGGAA----A-IVQTLEDTPFYARSLLSTTL 263
gi|653032131|ref|WP_027283892.      PPVSLPRT-LWRIRRPTRSEDGHA----R-VLRTLEDTPFYARSVIETRL 269
gi|378403832|emb|CCG08948.1|_1      PPAALPAGPSWGVARGIRSTG------PARVVETLEDTPFYTRSLVESVF 349
gi|754176764|ref|WP_041795395.      PPAALPAGPSWGVARGIRSTG------PARVVETLEDTPFYTRSLVESVF 265
gi|25989732|gb|AAN75036.1|_12-      PKAPLGA-TGWRVARGTRCDVG----APPTVLRTLEDTPFYARSLLSTRL 356
gi|860504149|ref|WP_048445338.      VPAPLPRT-LWRLPRTTRSDD-----GTAAVLRRFEDAPFYSRSLLSARI 262
gi|860474721|ref|WP_048434413.      LPAALPRT-LWRLPRATRSDD-----GQARVLRRFEDAPFYSRSLLAARI 262
gi|501291381|ref|WP_012333472.      IPAALPPT-LWRLKRATRSDD-----GQARVLRRFEDAPFYSRSQLAARI 262
gi|860453591|ref|WP_048427078.      VPAALPRT-LWRLPRATRSDD-----GQAALLRRFEDAPFYSRSLLSARI 249
gi|860560381|ref|WP_048464709.      VPAALPRT-LWRLERATRSDD-----GRAAVLRRFEDAPFYSRSLLSARI 249
```

```
gi|860527577|ref|WP_048454269.      VPAALPRT-LWRLPRATRSDD-----GRAALLRRFEDAPFYSRSLLAARI 262
gi|760871815|dbj|BAQ48812.1|_1      VPAALPRT-LWRLERATRSDD-----GQASVLRRFEDAPFYSRSLLSARI 249
gi|373567336|gb|EHP93308.1|_6-      LAAGLPSTGLWRMPRTTRGDD-----GRAQVIRTFEDTPFYARSLLASTL 270
gi|240009448|gb|ACS40674.1|_4-      LAAGLPSTGLWRMPRTTRGDD-----GRAQVIRTFEDTPFYARSLLASTL 261
gi|218523206|gb|ACK83791.1|_4-      LAAGLPSTGLWRMPRTTRGDD-----GRAQVIRTFEDTPFYARSLLASTL 261
gi|254269168|emb|CAX25134.1|_4      LAAGLPPTDLWRMPRTTRSDD-----GRAQVIRTFEDTPFYARSLLASTL 261
gi|163663749|gb|ABY31116.1|_2-      LAATLPPTGLWRMPRRSRSDD-----GRAQVIRTFEDTPFYARSLLASTL 264
gi|494837541|ref|WP_007563641.      LAAPLPSTSFWRMPRETRSDD-----GRARVLTTFEDTPFYSRSLLASSL 250
gi|914802769|ref|WP_050733395.      LAAPLPSTSFWRMPRETRSDD-----GRARVLTTFEDTPFYSRSLLASTL 250
gi|657246165|ref|WP_029357447.      PGATLPATRFWRMPRETRSDG-----GRSRVLATYEDTPFYARSLLASTL 250
gi|501276758|ref|WP_012319776.      PGATLPATRFWRMPRETRSDG-----GRSRVLATYEDTPFYARSLLASTL 250
gi|640229117|ref|WP_024829694.      PGATLPATRFWRMPRETRSDG-----GRARVLATYEDTPFYARSLLASTL 250
gi|518936109|ref|WP_020091984.      PGAALPATRFWRMPRETRSDG-----GRSRVLATYEDTPFYARSLLASTL 250
gi|760075276|ref|WP_043757989.      PAASLPATGFWRMPRETRSDD-----GRSRVLATYEDTPFYARSLLASTL 250
gi|759662308|ref|WP_043379914.      PAASLPATGFWRMPRETRSDD-----GRSRVLATYEDTPFYARSLLASTL 250
gi|498368472|ref|WP_010682628.      TAASLPATSFWRMGRETRSDD-----GRARVLATYEDTPFYARSLLASTL 250
gi|497224411|ref|WP_009538673.      PPVMLPKTRLWRIRRASRSQAP----EAARVAGTLEDTPFYARSLLSLRL 265
gi|589291345|gb|AHK79488.1|_12      PAHGLPTARIWRAPRATRADAGS-----ATIRHTLEDTPFYARSLVDARI 309
gi|644125233|ref|WP_025281955.      PAHGLPTARIWRAPRATRADAGS-----ATIRHTLEDTPFYARSLVDARI 254
gi|496218797|ref|WP_008932834.      RRQGLPTSAWWRIDRATRADDGR-----AAVRQTLTDTPFYVRSLLDTRI 268
gi|495659402|ref|WP_008383981.      PPVMLP-ASAWRVPRETRADPGTA----VTIVDTLEDTPFYARSLITGTL 263
gi|687960265|gb|KGE05197.1|_16      PRQQLPRASIWRMARSTQADRDTP----VTVQRTLEDTPFYSRSLLQYRA 270
gi|496312569|ref|WP_009021747.      PSTALPSTGIWRVARRMPGAE-----GKPAKLETLEDTPFYARSLIETTF 297
gi|495571757|ref|WP_008296336.      DAAALPRTGIWRVHRRMPGAE-----ARPTRLQTLEDTPFYARSLIKTEF 273
gi|908380581|ref|WP_049751705.      DPAPLP-TALWGIRRRIPSDTP----EKARLIETFEDTPFYARSLVATEV 254
gi|658524263|ref|WP_029718812.      PRQPLPTT-LWRIGRTMRTEPGV----PAVVEQTLEDTPFYARSMVRSGL 249
gi|332109922|gb|EGJ10550.1|_1-      PRQPLPTT-LWRIGRTMRTEPGV----PAVVEQTLEDTPFYARSMVRSGL 202
gi|760103377|ref|WP_043785587.      PRQPLPTT-LWRIDRTMRTEPGV----PAFVEQTLEDTPFYARSMVRSGL 249
gi|760129436|ref|WP_043810804.      PRQPLPTT-LWRIDRTMRTEPGV----PALVEQTLEDTPFYARSMVRSGL 249
gi|7416798|dbj|BAA94047.1|_1-4      PRQPLPTT-LWRIDRTMRTEPGV----PAFVEQTLEDTPFYARSMVRSGL 372
gi|75345570|sp|P95619.1|CRTC_R      PRQPLPTT-LWRIGRTMRTEPGV----PALVEQTLEDTPFYARSMVRSGL 372
gi|913634290|gb|KNZ32951.1|_1-      PRQALPRT-LWRIGRTMRTDPGV----PATVTRTLEDTPFYARSMLRSGL 254
gi|809277751|ref|WP_046331236.      ERVSLRKT-GWGIKRNMRSEKTG----TVELLNTYEDTPFYARSRIQSHL 255
gi|518258896|ref|WP_019429104.      PVQRLPKT-AWRIDRRMRSTQ------AVRVHEQLEDTPFYQRALLQFDY 253
gi|518255752|ref|WP_019425960.      PVQNLSKT-GWRIDRRMRSTQ------AVRVHEQLEDTPFYQRALLQFDH 253
gi|918152310|gb|KOF08324.1|_1-      PVQALPKT-GWRINRRMRSAN------AVRVQEQLEDTPFYQRALLQFNF 253
gi|61653243|gb|AAX48196.1|_1-2      PVQRLPKT-GWRIDRRMRSEQ------PVQVQEQLEDTPFYQRALLQFDH 253
                                        *     *   *                   ::*** *: :

gi|350284778|gb|AEQ27763.1|_1-      LDRPVTAVHESLSLDRFRSRWVQHLLPYRMPRR- 403
gi|521992738|ref|WP_020504009.      LDRPVTAVHESLSLDRFRSRWVQHLLPYRMPRR- 301
gi|557030915|gb|ESQ08988.1|_1-      LDRPLQAVHESLSLDRFRSPIVQHMLPYRMPRH- 292
gi|494353717|ref|WP_007191968.      QGRPIEAVHESLSLDRFRSAWVQHLLPYRMPRR- 292
gi|570733543|gb|AHF04706.1|_24      QGRQAEAVHESVSLDRFTSPWVQPLLPYRMPKR- 312
gi|760081160|ref|WP_043763735.      QGRQAEAVHESVSLDRFTSPWVQPLLPYRMPKR- 312
gi|528190751|gb|EPY01080.1|_43      FGEKVTGFHESLCLDRFRSGWVQALLPFRMPRR- 347
```

```
gi|748594904|ref|WP_039853084.      FGEKVTGFHESLCLDRFRSGWVQALLPFRMPRR- 300
gi|488815067|ref|WP_002727473.      FGEEVTGFHESLCLDRFRTGWVQALLPFRMPRR- 385
gi|505094729|ref|WP_015281831.      LGKPVTGVHESLSLDRFRQGWVQMLLPFRMPRLA 289
gi|754165669|ref|WP_041786299.      AGQDVTAIHESLSLDRFDSTWVQVLLPFRMPRLA 310
gi|739596279|ref|WP_037453842.      GSRPATAIHESLSLDRFRAGWVQAMLPFRMPR-- 287
gi|764363842|ref|WP_044336870.      FGEDAQAVHESLSLGRFRSPIVQSMLPYRMPR-- 302
gi|644239143|ref|WP_025293539.      FGEDVQAVHESLSLGRFRSPIVQSMLPYRMPR-- 302
gi|503035479|ref|WP_013270455.      LGEACEGVHESLSLGRLRSPLVRAMLPFRMPR-- 289
gi|617472745|gb|KAI93218.1|_2-      LGEPVHAMHESVSLDRFRKRWVQSLLGWRMPR-- 375
gi|737603811|ref|WP_035574467.      YGQDTTAVHESLSLVRFRTRWVKLLLPFRMPRRA 287
gi|648530966|ref|WP_026222717.      RGETIHWVNESLSLDRFANPLIRLMLPFRMPRRA 284
gi|288897641|gb|ADC63477.1|_28      LGQRATSVHESLCLDRFSTQWVKVLLPFRMPR-- 375
gi|760114352|ref|WP_043796307.      LGQRATSVHESLCLDRFSTQWVKVLLPFRMPR-- 287
gi|494098690|ref|WP_007039487.      LGEHATSVHESLCLDRFSSTWVRTLLPFRMPRIA 291
gi|75388571|sp|Q7X3G5.1|CRTC_T      AGETATCVHESLSLDRFASPVVQLMLPFRMPR-- 376
gi|390426908|gb|AFL73973.1|_35      LGEPVTAMHESVCLDRFDSRWVQVLLPFRIPR-- 336
gi|752807498|ref|WP_041446922.      LGEPVTAMHESVCLDRFDSRWVQVLLPFRIPR-- 297
gi|760074757|ref|WP_043757486.      LGEPVTAMHESVCLDRFNSRWVQTLLPFRIPR-- 305
gi|918211006|ref|WP_052348303.      LGEPVTAMHESVCLDRFNSRWVQTLLPFRIPR-- 297
gi|499982040|ref|WP_011662758.      LGETATAMHESLCLDRFNSRWVQTLLPFRIPR-- 305
gi|493840365|ref|WP_006787509.      FGEPVTAMHESLDLDRYSKKWVRSLMPFRMPR-- 290
gi|653037601|ref|WP_027289289.      AGEPVRAMHESLSLTRFDTRIVRLMLPFRMPR-- 314
gi|519009821|ref|WP_020165696.      LGEPVHGVHESLDLERFRSGWVQMLLPFRMPRRS 310
gi|518759539|ref|WP_019916969.      LGEPVHGVHESLDLERFRSGWVQMLLPFRMPRRS 306
gi|495340016|ref|WP_008064750.      LGEPVHGVHESLDLERFSSGWVQMLLPFRMPRRS 306
gi|517223140|ref|WP_018411958.      LGEPVRGVHESLDLTRFSSGWVQMLLPFRMPRRA 308
gi|889919304|ref|WP_048861895.      DGEPVIAMHEALSMRRFVAPWVQAMLPFRMPR-- 303
gi|496441596|ref|WP_009150441.      LGEPVTAVHESLSLDRFAQPWVRMLLPFRMPR-- 384
gi|146409846|gb|ABQ38352.1|_10      GGEDVAVMHESLSLDRFKQPIVQAMLPFRMPR-- 392
gi|908659074|ref|WP_049795525.      GGEDVAVMHESLSLDRFKQPIVQAMLPFRMPR-- 299
gi|365289544|emb|CCD93979.1|_1      QGEDVAVMHESLSLDRFQMPIVQAMLPFRMPRRA 394
gi|41018902|gb|AAR98493.1|_10-      HGEDVAVMHESLSLDRFQMPIVQAMLPFRMPR-- 392
gi|653027709|ref|WP_027279528.      LGEQTTLMHESLSLERFRMPVVQAMLPFRMPRR- 302
gi|499791021|ref|WP_011471755.      FGEHATLMHESLSLDRFKMPVVQAMLPFRMPRR- 302
gi|499823412|ref|WP_011504146.      LGEPVTLVHESLSLDRFRMPIVQAMLPFRMPRR- 302
gi|499762149|ref|WP_011442883.      LGERVTLMHESLSLDRFKMPVVQAMLPFRMPRR- 302
gi|829076993|ref|WP_047310044.      LGEKVTLMHESLSLDRFRMPVVQAMLPFRMPRR- 302
gi|503269006|ref|WP_013503667.      LGEKVTLMHESLSLDRFRMPVVQAMLPFRMPRR- 302
gi|499470438|ref|WP_011157078.      LGEKVTLVHESLSLDRFRMPVVQAMLPFRMPRR- 302
gi|501486651|ref|WP_012495141.      LGEKVTLVHESLSLDRFRMPVVQAMLPFRMPRR- 302
gi|550975051|ref|WP_022723265.      LGEPVTLMHESLSLDRFKMPVVQAMLPFRMPRR- 302
gi|764538658|ref|WP_044412067.      LGEKVTLMHESLSLDRFKLPVVQAMLPFRMPRR- 302
gi|754113006|ref|WP_041748751.      LGEPVTLMHESLSLDRFKIPIVQAMLPFRMPR-- 312
gi|737680904|ref|WP_035649991.      LGKPVTLIHESLSLDRFKMPLVQAMLPFRMPR-- 306
gi|503405597|ref|WP_013640258.      GGEPVEAVHETLDCDRLVHPAVQFMLPFRMPRR- 305
gi|657196689|ref|WP_029312388.      DGERLDGVHETLDLDRFTMPVVQAMLPFRMPRR- 307
```

```
gi|703385326|ref|WP_033417843.      CGERTTGVHESLDLDRFRQPVVQWMLPFRMPR-- 295
gi|653032131|ref|WP_027283892.      LGERATAVHESLDLDRFAHPSMYAMLPFKVPR-- 301
gi|378403832|emb|CCG08948.1|_1      HGETVRSVHESLDLDRLTSRWVPWLLPFRMPRR- 382
gi|754176764|ref|WP_041795395.      HGETVRSVHESLDLDRLTSRWVPWLLPFRMPRR- 298
gi|25989732|gb|AAN75036.1|_12-      AGEETRAVHESIDLDRLRAGWVWPLLPFRMPRR- 389
gi|860504149|ref|WP_048445338.      CGEAVQPVHEALCLDRFSHPLVRLMLPFRMPR-- 294
gi|860474721|ref|WP_048434413.      CGEAVEPVHEALDLDRFRNPLVRAMLPFRMPR-- 294
gi|501291381|ref|WP_012333472.      CGEAVEPVHEALCLDRFRHPLVRLMLPFRMPR-- 294
gi|860453591|ref|WP_048427078.      CGEAVRPVHESLDCDRLTHPLVRFMLPFRMPR-- 281
gi|860560381|ref|WP_048464709.      CGEAVQPVHESLDLDRLTHPLVRFMLPFRMPR-- 281
gi|860527577|ref|WP_048454269.      CGEAVRPIHESLDLDRLTHPLVRFMLPFRMPR-- 294
gi|760871815|dbj|BAQ48812.1|_1      CGEAVEPVHESLDLDRLTHPLVRFMLPFRMPR-- 281
gi|373567336|gb|EHP93308.1|_6-      DGEAVQPVHESLSLTRFRNPLVRLMLPFRMPRR- 303
gi|240009448|gb|ACS40674.1|_4-      DGEAVQPVHESLSLTRFRNPLVRLMLPFRMPRR- 294
gi|218523206|gb|ACK83791.1|_4-      DGEAVQPVHESLSLTRFRNPLVRLMLPFRMPRR- 294
gi|254269168|emb|CAX25134.1|_4      DGEAVQPVHESLSLTRFRNPLVRLMLPFRMPRR- 294
gi|163663749|gb|ABY31116.1|_2-      DGEAVQPVHESLSLTRFRNPLVRLMLPFRMPRR- 297
gi|494837541|ref|WP_007563641.      CGEPVRAVHESLSLDRFRNPLVQLMLPFRMPR-- 282
gi|914802769|ref|WP_050733395.      AGEPVRAVHESLSLDRFRNPLVQLMLPFRMPR-- 282
gi|657246165|ref|WP_029357447.      CGEPVRAMHESLSLDRFRNPMVRLMLPFRIPR-- 282
gi|501276758|ref|WP_012319776.      CGEPVRAMHESLSLDRFRNPMVRLMLPFRIPR-- 282
gi|640229117|ref|WP_024829694.      CGEPVRAMHESLSLDRFRNPMVRLMLPFRIPR-- 282
gi|518936109|ref|WP_020091984.      GGEPVRAVHESLSLDRFRNPMVRLMLPFRIPR-- 282
gi|760075276|ref|WP_043757989.      GGEPVRAMHESLSLDRFRNPLVRLMLPFRIPR-- 282
gi|759662308|ref|WP_043379914.      GGEPVRAMHESLSLDRFRNPLVRLMLPFRIPR-- 282
gi|498368472|ref|WP_010682628.      CGEPVRAMHESLSLDRFRNPLVQLMLPFRMPR-- 282
gi|497224411|ref|WP_009538673.      AGEDATAVHESLDMRRFTNPLVQAMLPFRMPR-- 297
gi|589291345|gb|AHK79488.1|_12      HGEKAVAFHESLSLNRFESPVVQLMLPFRMPRRA 343
gi|644125233|ref|WP_025281955.      HGEKAVAFHESLSLNRFESPVVQLMLPFRMPRRA 288
gi|496218797|ref|WP_008932834.      EGEPGLAFHESLSLERFETTVVQLMLPFRMPRRA 302
gi|495659402|ref|WP_008383981.      GGEPVTAVHESLSLDRFAAPWVQAMLPF------ 291
gi|687960265|gb|KGE05197.1|_16      LGEPVRAVHESLNLDRFSRRWVQLLLPFRLPRRA 304
gi|496312569|ref|WP_009021747.      GGEPGAGIHESLSMQRFEQRWVQTLLPFRMPR-- 329
gi|495571757|ref|WP_008296336.      GGETGTGIHESLSMERFEQRWVQTLLPFRMPRRT 307
gi|908380581|ref|WP_049751705.      DGRPLHAVHESLNLDRFASAWVKPLLPFRMPRR- 287
gi|658524263|ref|WP_029718812.      LGEVVTSVHETMLLPRVITLPVRLMLPWRMPRR- 282
gi|332109922|gb|EGJ10550.1|_1-      LGEVVTSVHETMLLPRVITLPVRLMLPWRMPRR- 235
gi|760103377|ref|WP_043785587.      LGEVVTSVHETMLLPRVITLPVRLMLPWRMPRRS 283
gi|760129436|ref|WP_043810804.      LGEVVTSVHETMLLPRVITLPVRLMLPWRMPRRS 283
gi|7416798|dbj|BAA94047.1|_1-4      LGEVVTSVHETMLLPRVITLPVRLMLPWRMPRRS 406
gi|75345570|sp|P95619.1|CRTC_R      LGEVVTSVHETMLLPRVITLPVRLMLPWRMPRRA 406
gi|913634290|gb|KNZ32951.1|_1-      LGEAVESVHETLSVPRLVSPAVRFMLPWRMPR-- 286
gi|809277751|ref|WP_046331236.      LGEGVISMHETLNVNRLKSNIVQLMLPWRMPRN- 288
gi|518258896|ref|WP_019429104.      AGESLLAFHETLSVPRLVSPVVQAMLPWRMPRRA 287
gi|518255752|ref|WP_019425960.      AGEPLLAFHETLSVPRLVSPVVQAMLPFRMPRRA 287
gi|918152310|gb|KOF08324.1|_1-      AGQHLLAFHETLSVPRLVSPVVQAMLPWRMPRRA 287
```

```
gi|61653243|gb|AAX48196.1|_1-2      AGEPLLAFHETLSVPRLVSPVVQAMLPWRMPRRA 287
                                     ..    .:*::   *     :  :: :
```

PLEASE NOTE: Showing colors on large alignments is slow.

1. Importance of the N-terminal part of CrtC

In our previous study we have already shortly discussed the preliminary results on the importance of the N-terminal part of CrtC for the catalytic activity (Hiseni et al. 2011). In order to investigate this further, truncated (*Tr*- and *Rg*CrtC) and N-terminal point mutants (*Tr*CrtC) were constructed (Table S1). We have shown here that the activity of the truncated versions is fully retained (Figure 6, main text). It should be noted here that the truncated version of *Tr*CrtC shows a larger molecular weight (Figure S2, lane 2) compared to the native 'cleaved' form (Figure S2, lanes 3,4), which is due to the attached N-terminal His-Tag in all constructs that is cleaved off from the full length protein, resulting in a smaller protein.

Table S1. N-terminal mutants of *Rubrivivax gelatinosus* (*Rg*CrtC) and *Thiocapsa roseopersicina* (*Tr*CrtC) carotenoid 1,2-hydratase.

**Name**	**Mutation**
*Rg*CrtC truncated	*Rg*CrtC without first 45 AAs
*Tr*CrtC truncated	*Tr*CrtC without first 57 AAs
*Tr*CrtC S58V	*Tr*CrtC bearing mutation S58V
*Tr*CrtC S58Q	*Tr*CrtC bearing mutation S58Q

Apart from expression levels, the activities of crude cell extracts of the N-terminal constructs were measured with lycopene as substrate. Since crude cell extracts were used, the results cannot be quantitated. However, in combination with the expression levels as shown in Figure S2, indicative conclusions can be drawn. As stated in our previous study, the N-terminus is not important for catalytic activity (Hiseni et al. 2011). This again was proven here, as the truncated *Rg*- as well as *Tr*CrtC were active. Furthermore, although the introduction of N-terminal point mutations did affect the cleavage rate of the N-terminal part, it did not affect the activity of *Tr*CrtC.

Figure S2. SDS-PAGE (10%) analysis of expression of N-terminal mutants from *T. roseopersicina* CrtC (*Tr*CrtC). M, Precision plus protein standard (BioRad). First lane of each sample shows cells before induction with 0.1 mM IPTG and the second lane shows cells after overnight expression at 25°C. 1, *Tr*CrtC wild type; 2, *Tr*CrtC truncated; 3, *Tr*CrtC S58V; 4, *Tr*CrtC S58Q.
Full length protein = 44 kDa, truncated protein w/o His-tag = 38 kDa, truncated protein with His-tag = 39 kDa.

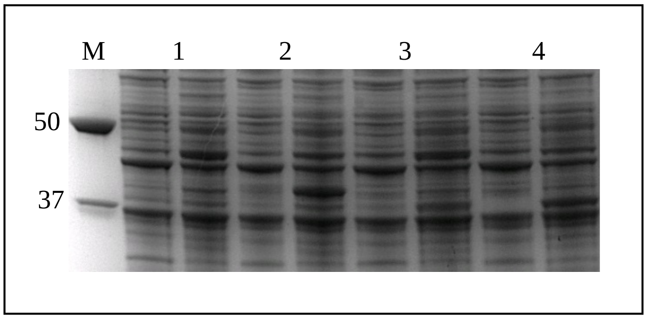

Although the reason for the truncation is still unknown, and the N-terminal region is not involved in the catalytic activity, we did explore this phenomenon a bit further. The cleavage site was determined by MS analysis, and appeared between S57 and S58. To determine whether the truncation of recombinant *Tr*CrtC was processed by *E. coli* endogenous proteases or the enzyme itself, two mutants at the first amino acid after cleavage were produced. The S58 position was modified by substitutions with valine (same size, different chemical features) or glutamine (different size, same chemical features), which are no targets for known *E. coli* proteases. All resulting proteins were cleaved, but at a different rate. It seems that the cleavage rate increased in the order of wild type < S58V < S58Q. This conclusion is based on the fact that in the wild type sample in Figure S2 mainly the 44 kDa band is visible (lane 1). In the S58V mutant about the same amount of both, the 38- as well as the 44 kDa bands could be detected (lane 3), while in the S58Q mutant mainly the 38kDa band could be identified (lane 4). These differences in cleavage rate might be explained by the fact that the substitution of an amino acid by a larger or more hydrophobic amino acid could result in conformational changes which promote the processing activity, either by host proteases or through self-cleavage. Since this truncation was not the focus of this study, we did not pursue this any further. Nevertheless, additional experiments are needed to get more insight into this occurrence. For example, by expressing larger amounts of the corresponding proteins, and quickly purifying them, one could follow the change of the protein size in time. With the wild type *Tr*CrtC, we have already seen that even though the purified enzyme consisted of both sizes proteins, after few days of storage, the 44 kDa could not be detected anymore, pointing towards self-cleavage (Hiseni et al. 2011), which is not disturbed by the mutations prepared in this study.

2. Homology model of CrtC

In order to investigate which one of the fully conserved residues could be involved in the catalytic hydration reaction, we tried to build a 3D structure model of *Tr*CrtC. The CrtC secondary structure prediction was carried out with the program PolyView 2D (Porollo et al. 2004). Then the SWISS-MODEL program was used to model the structure of *Tr*CrtC (Arnold et al. 2006). All programs were used from their respective website, using the default settings.

Figure S3. A homology model of CrtC from *T. roseopersicina* based on the crystal structure of a putative AttH (PDB id: 2ICH) from *Nitrosomonas europaea*. The ribbon diagrams depict front (left-hand side) and back (right-hand side) view of CrtC. The structure is color-coded from the N-terminus (blue) to the C-terminus (red).

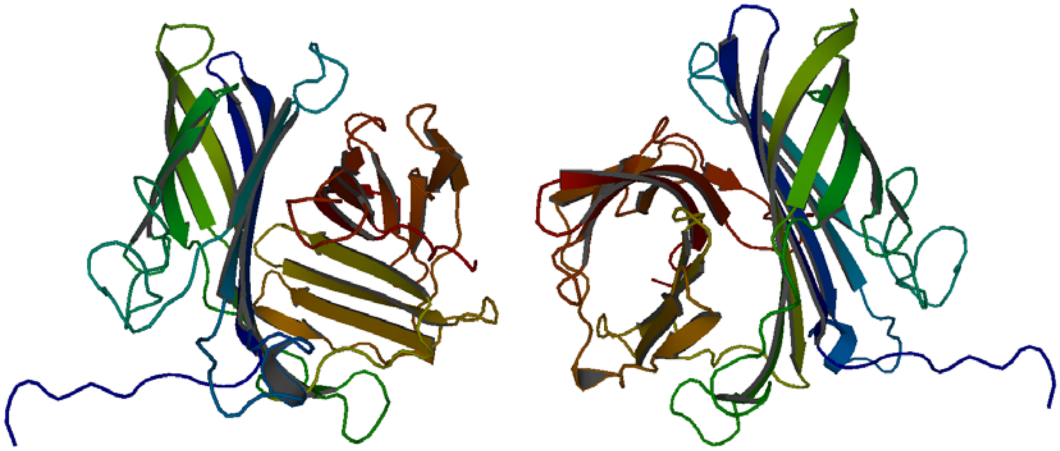

The homology model was based on the only known 3D structure (PDB 2ICH) that showed some sequence identity (15.4%) to the CrtC (Figure S3), which is generally regarded as too low to build a good model. Modelled residue range was between 95 and 342 (QMEAN *Z*-Score: -6.054). The homologue is the putative AttH protein from *Nitrosomonas europaea* (Chiu et al. 2010). It belongs to a protein family of unknown function (DUF2006), which has remote similarity to the family PF07143 encompassing carotenoid 1,2-hydratases. The topology of the CrtC model structure shows similarity to lipocalins, proteins that bind and transport small hydrophobic molecules (Flower 1996). The lipocalin fold is typically formed by a large, twisted beta-sheet that closes in the back to form a central, internal, ligand-binding cavity. This folding motif is frequently found in porins, transmembrane proteins or in general in proteins that bind hydrophobic ligands/substrates (LaLonde et al. 1994).

Figure S4. CrtC conserved residues. Ribbon diagram of *Tr*CrtC with marked regions that contain highly conserved amino acid residues. The sequence motifs, which correspond to those regions, are shown in boxes (I – VI). N and C indicate N-and C-terminus, respectively.

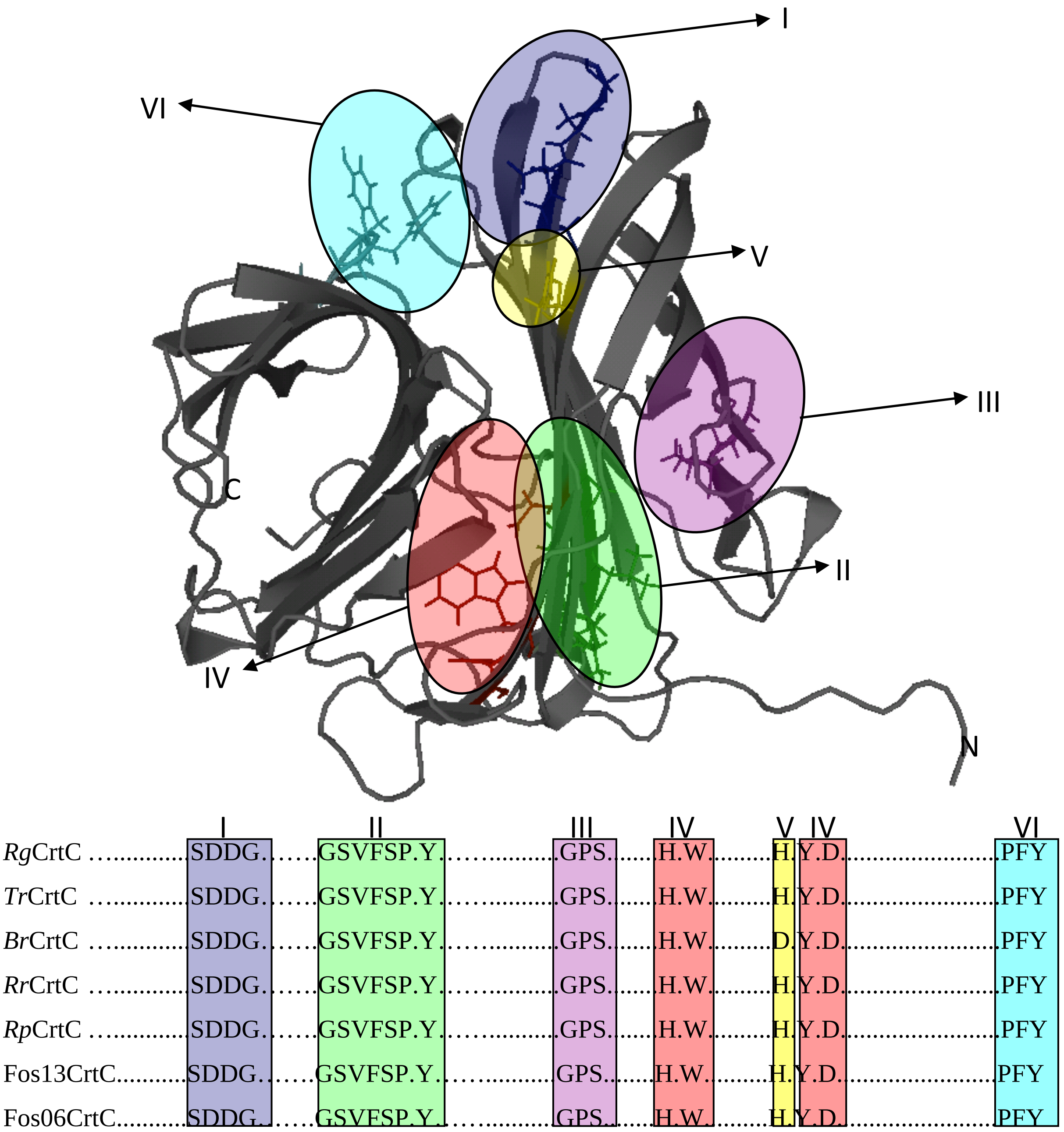

We visualized the conserved residues onto the model to investigate if there is any relationship between the conserved regions and specific locations in the homology model of CrtC (Figure S4). Six striking sequence motifs were selected (I – VI) and the results show that, indeed, most of the highly conserved residues that were found across all (putative) CrtC's (Figure S1) are located either at the bottom or upper part of the cavity, with one motif (III) located outside the cavity. From a catalytic point of view, the amino acids located in regions IV and V, i.e. aspartic acid (D), tyrosine (Y) and histidine (H), are most probably involved in the catalytic hydration, as these amino acids are commonly involved as active residues in acid-base type catalyzed reactions in the active sites of enzymes (Bartlett et al. 2002). Furthermore, in this model they are all aligned and in close distance to each other (Figure S5), which is important for the contact with the substrate.

Figure S5. View of the potential active site of *Tr*CrtC. The conserved residues H237 (yellow), W239 (red), Y264 (blue) and D266 (green) are show as sticks, with hydrogen bond as yellow dots (region IV).

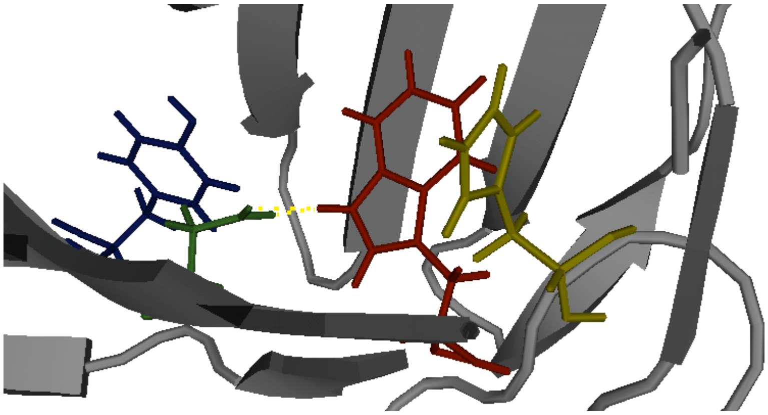

Although the model is based on very low homology, this structure is in agreement with our final hypothesis, where Y266 could make a bond to D268 through a water molecule, and H239 is in H-bridging distance (Figure S5), so all amino acids are correctly placed to assume their hypothetical role (Figure 8, main text). Interestingly, according to the homology model, the residues H264 (*Rg)* and H262 (*Tr*), which mutation to Ala does not impair the activity of the enzyme so much, are located away (region V) from the potential active site residues. This observation further allows our hypothesis that region IV is the active site of CrtC. On the other hand, the three totallly conserved hydrophobic residues proline, phenylalanine and tyrosine in region VI might play the role of attracting the hydrophobic substrate and placing it in the right position. The highly conserved mainly hydrophobic amino acids in region II, which are located close to potential active side residues in region IV, might play a role in stabilization of the substrate during catalytic activity.

Conclusion

The homology model built for this study is merely a working model, given the limited level of sequence identity. However, the position of key residues correspond to the activity data of the mutants and allows our hypothesis on the hydration mechanism of CrtC. Due to this fact that the preserved active site residues do end up in close proximity to each other (see figure S5), we consider this model to provide a good basis for future studies, although a real crystal structure is of the utmost importance to confirm our hypothesis.

Acknowledgements

We thank Jan van Leeuwen (Groningen University) for making the CrtC models.

References

Arnold K, Bordoli L, Kopp J, Schwede T (2006) The SWISS-MODEL workspace: a web-based environment for protein structure homology modelling. Bioinformatics 22(2):195-201. doi:10.1093/bioinformatics/bti770

Bartlett GJ, Porter CT, Borkakoti N, Thornton JM (2002) Analysis of catalytic residues in enzyme active sites. J Mol Biol 324(1):105-121. doi:10.1016/S0022-2836(02)01036-7

Chiu H-J, Bakolitsa C, Skerra A, Lomize A, Carlton D, Miller MD, Krishna SS, Abdubek P, Astakhova T, Axelrod HL, Clayton T, Deller MC, Duan L, Feuerhelm J, Grant JC, Grzechnik SK, Han GW, Jaroszewski L, Jin KK, Klock HE, Knuth MW, Kozbial P, Kumar A, Marciano D, McMullan D, Morse AT, Nigoghossian E, Okach L, Paulsen J, Reyes R, Rife CL, van den Bedem H, Weekes D, Xu Q, Hodgson KO, Wooley J, Elsliger M-A, Deacon AM, Godzik A, Lesley SA, Wilson IA (2010) Structure of the first representative of Pfam family PF09410 (DUF2006) reveals a structural signature of the calycin superfamily that suggests a role in lipid metabolism. Acta Crystallogr, Sect F: Struct Biol Cryst Commun 66(10):1153-1159. doi:10.1107/S1744309109037749

Flower DR (1996) The lipocalin protein family: Structure and function. Biochem J 318(1):1-14. doi:10.1042/bj3180001

Hiseni A, Arends IWCE, Otten LG (2011) Biochemical characterization of the carotenoid 1,2-hydratases (CrtC) from *Rubrivivax gelatinosus* and *Thiocapsa roseopersicina*. Appl Microbiol Biotechnol 91(4):1029-1036. doi:10.1007/s00253-011-3324-1

LaLonde JM, Bernlohr DA, Banaszak LJ (1994) The up-and-down beta-barrel proteins. FASEB J 8(15):1240-7.

Porollo AA, Adamczak R, Meller J (2004) POLYVIEW: A flexible visualization tool for structural and functional annotations of proteins. Bioinformatics 20(15):2460-2462. doi:10.1093/bioinformatics/bth248
